# Activating effective functional hand movements in individuals with complete tetraplegia through neural stimulation

**DOI:** 10.1038/s41598-022-19906-x

**Published:** 2022-10-06

**Authors:** Christine Azevedo Coste, Lucie William, Lucas Fonseca, Arthur Hiairrassary, David Andreu, Antoine Geffrier, Jacques Teissier, Charles Fattal, David Guiraud

**Affiliations:** 1 CAMIN, INRIA, University of Montpellier, Montpellier, France; 2 NEURINNOV, Montpellier, France; 3 University of Montpellier, Montpellier, France; 4 APHP, Paris/CHU Rennes, Rennes, France; 5 ORTHOSUD, Saint Jean Clinic, Saint-Jean-de-Védas, France; 6 Center Bouffard-Vercelli USSAP, Perpignan, France

**Keywords:** Translational research, Biomedical engineering

## Abstract

Individuals with complete cervical spinal cord injury suffer from a permanent paralysis of upper limbs which prevents them from achieving most of the activities of daily living. We developed a neuroprosthetic solution to restore hand motor function. Electrical stimulation of the radial and median nerves by means of two epineural electrodes enabled functional movements of paralyzed hands. We demonstrated in two participants with complete tetraplegia that selective stimulation of nerve fascicles by means of optimized spreading of the current over the active contacts of the multicontact epineural electrodes induced functional and powerful grasping movements which remained stable over the 28 days of implantation. We also showed that participants were able to trigger the activation of movements of their paralyzed limb using an intuitive interface controlled by voluntary actions and that they were able to perform useful functional movements such as holding a can and drinking through a straw.

## Introduction

The incidence of spinal cord injuries (SCIs) in Western Europe and the United States is estimated at 16 and 40 cases *per* million, respectively^1^. SCIs can have a devastating impact on patient health, autonomy and quality of life. Technical aids (e.g., motorized wheelchairs, orthoses, medical electric beds, transfer boards, home automation, etc.) can restore some independence to people with tetraplegia, but recovering upper limb functions is still the priority for functional recovery expressed by patients^2–6^. Indeed, most of the activities of daily living are performed via hand movements and therefore the restoration of active motor skills in the forearm, hand and wrist would allow for greater autonomy and thus increased quality of life. In the absence of spinal cord repair solutions, only partial answers are available today. We thus proposed a breakthrough innovation based on selective neural stimulation which, to date, is the first one to induce synergic hand movements with only one electrode on the median nerve and one electrode on the radial nerve. Indeed, other approaches provide either partial or much more cumbersome solutions. Functional surgery is commonly used^7,8^ and more recently, nerve transfers have been attempted to re-innervate paralyzed muscles to regain voluntary control of the hand^9,10^. However, both methods require a sufficient number of muscles or nerves that are still under voluntary control. The transferred muscles and the remaining agonist muscles must also be strong enough to ensure effective recovery^11,12^. A part of the tetraplegic population is therefore not eligible for conventional functional surgery. The alternative is to use technical aids based on functional electrical stimulation (FES) or orthoses^13^.

FES alone, implanted or external, can be used efficiently, provided that the sub-lesional paralyzed muscles are still innervated by intact motoneurons^14^. One of the first applications of FES to recover hand motion was reported by Catton and Backhouse in 1954. FES was subsequently used to recover grasping movements in patients with high tetraplegia as early as 1963^15–17^. These devices used intramuscular or epimysial electrodes, requiring one electrode for each muscle involved in the target movement. Noninvasive surface FES can also provide hand movements but with limited access to deep muscles or to muscles dedicated to the thumb (e.g. *Abductor Policis Brevis*). Surface electrodes need for an accurate placement from day to day to achieve a reliable functional movements without recalibration. Finally the surface electrode’s placement are subject to skin relative movements. To overcome partly these problems, fixing the electrodes on the garment was proposed^14^. However most of external FES devices failed to be used at a large scale due to the garment rigidity, lack of personalization or limitation to groups that either incomplete or with wrist control^18^. So these devices are rather used for rehabilitation and reinforcement^14^ without new devices as reported in a very recent review^19^. Surface electrode arrays^20,21^ can provide a more flexible and larger set of functional movements but they are clearly limited, in their current form, to a laboratory use as it demands for a day to day calibration under the supervision of a skilled physiotherapist. Ajiboye et al.^22^ also provided a rich set of movements but through a highly invasive percutaneous set of electrodes (#36) that could hardly be translated into a wide clinical practice. Both, external or percutaneous devices are very limited in terms of acceptability, safety and efficiency and thus are not used by patients in a daily-living context even though a rich repertoire of movements can be achieved. The only widely used successful device that has been proposed was the FreeHand®: more than 250 patients^23^ have had it successfully implanted with clear benefits, proving the interest of such a technological solution for recovering hand movements^24^. Up to 12 muscular electrodes have been implanted to activate various hand tasks. A research version attempted to replace several muscle electrodes with a single neural 4-contact electrode^25^. During an intraoperative acute testing within a scheduled surgery, the results on selectivity remained limited, due to the adopted approach which was based on a monopolar scanning of the different electrode contacts. The same team tried on 2 patients to use epineural electrodes to activate a greater number of movements in the whole upper limb. Indeed, 6 epineural electrodes were added to the intramuscular electrodes (14 on patient 1 and 15 on patient 2) leading to a very cumbersome setup with 2 Implanted Pulse Generators^26^*.* However, they further tested a simplified steering current paradigm with the epineural electrode and showed stability and increased selectivity compared to intramuscular stimulation^27^.

A step further would be to activate muscle groups via a limited number of epineural electrodes. Selective multicontact neural stimulation has the advantage of activating a large number of muscles via a limited number of electrodes, while requiring much less energy than epimysial or intramuscular stimulation and, by far compared to surface stimulation.

Human trials have already demonstrated the feasibility of this approach for restoring hand movements^25,28^ but, as it combines multisite neuromuscular stimulation, it is very complex to set up and is therefore no more advantageous than the original FreeHand system. The limited effectiveness of nerve stimulation is due both to the limited selectivity of the electrode used and to the simplicity of the stimulation paradigm: four contacts with a global reference away from the electrode, with only one of the four contacts being used during stimulation. More complex multi contact electrodes have been used successfully in the human upper limb, namely the FINE^29,30^ and the TIME^31,32^ electrodes. Very recently, fine hand movements were obtained in primate with TIME electrodes leading to a promising alternative yet to be proved in humans^33^. However, the stimulation paradigms remained limited to bipolar-like stimulation where a single active contact was used toward a global ground.

In previous theoretical and preclinical studies^34^, we have shown that optimized complex current distributions over multicontact epineural electrode poles lead to higher selectivity within target nerves. We have therefore successfully applied this approach to the human forearm in trials^35^ during which we performed intraoperative sessions by stimulating the median or radial nerve in eight subjects with tetraplegia during scheduled surgeries. We demonstrated that it was possible to obtain isolated muscle contractions for flexors or extensors (fingers, wrist, thumb) in most subjects. We also obtained compound movements that could be used to produce key grip, hook and palmar grips. However, the patients were under general anesthesia and only one nerve was evaluated in each surgery. In addition, the scanning of the intensities were limited to predefined values with a coarse step to limit the needed time. It prevented a fine exploration of the stimulation parameters.

The present work goes a step further through a short term clinical trial: on 2 participants with complete tetraplegia, we show for the first time that with only 2 multicontact epineural electrode cuffs associated with an intuitive user control interface, the participants were able to autonomously activate a functional grasping. These preliminary results are all the more encouraging since the performance was obtained in approximately 3 weeks.

## Results

Both participants had complete C4 AIS A tetraplegia. Two multicontact, self-adjusting epineural electrodes (CorTeC Gmbh, Freiburg, Germany) were wrapped around the target nerves above the elbow during a surgery under general anesthesia. The electrodes consisted of 2 outer rings and a central ring composed of equally spaced contacts (see “Materials” section). Depending on the electrode diameter, up to 6 (radial nerve) or 9 (median diameter) central contacts are available. The electrodes’ diameters are self-sizing so that depending on the actual diameter of the nerve, the winding is more or less large. Given the diameter of the nerves, the number of useful contacts was eventually limited to 8 for the median nerve and 6 for the radial nerve for both patients. This original and tailored design of the electrode is based on our previous preclinical and simulations studies^34,36^.

Each participant followed 3 experimental sessions per week, during 28 days. The first session was dedicated to electrode configuration and stimulation parameters tuning, the second session was dedicated to user interface adjustment and the last session was focused on functional tests of the optimized setup. The movements sought for gripping were the key grip, the palmar grip with the thumb and the opening of the hand. Concerning the selective configurations used, we tested 3 different current distributions: (1) Tripolar Longitudinal (TLR) configuration composed of a central contact as cathode and the two rings as anodes, (2) Steering Current (STR) for which a third anode was used on the opposite to the selected cathode, (3) Transverse Tripolar (TTR) for which 2 anodes were added to the TLR on each side of the selected cathode. Previous studies^35,36^ proved that the focus area of the activation under the cathode is the largest with TLR and the smallest with TTR. STR provides an intermediate focus. The Supplementary Material “Electrode’s configurations” section gives the detailed procedure.

### Selection of functional movements based on the synergistic muscles’ responses can be obtained

The selectivity search consists in exploring the muscles’ responses of the obtained movements while changing the cathodic contact and the configuration. This search is completed by an assessment performed with surface EMG. Indeed, recruitment curves are obtained varying the intensity in isometric conditions; the resulting M-waves allow us to objectively quantify these responses. The median nerve innervates predominantly the flexor muscles of the forearm and the hand. Surface EMG electrodes were placed on: the *flexor carpi radialis* (**FCR**) responsible for the wrist flexion, the *pronator teres* (**PT**) responsible for pronation of the forearm and the wrist, the *flexor digitorum superficialis* (**FDS**) responsible for the digit (except thumb) flexion, the *flexor pollicis longus* (**FPL**) responsible for the thumb flexion and the *abductor pollicis brevis* (**APB**) responsible for the thumb abduction. The radial nerve provides motor innervation to muscles in the arm and forearm that are mostly extensors. Surface EMG electrodes were placed on: the *extensor carpi radialis* (**ECR**) responsible for the wrist extension, the extensor pollicis longus (**EPL**) responsible for the thumb extension and the *extensor digitorum communis* (**EDC**) responsible for the fingers extension.

Considering the large set of possibilities—for the median nerve 8 contacts for 3 configurations would lead to 24 sessions while varying the intensity, the pulse-width and eventually the frequency—it was necessary to sort the configurations in order to select the more appropriate one for each desired movement. There were two options: (i) using low current intensities to activate isolated muscle contractions but with a limited strength and combine these individual activations, (ii) selecting the configurations that induce synergistic muscle activations producing global functional movements. Even though the first approach was first envisioned, the second one was the only feasible way to achieve optimal configurations. For the grasping movements, we tried to favor **FDS/FPL/APB** contractions, to avoid wrist flexion/pronation, so a relevant subgroup of contacts was selected using TLR configuration explorations only with a fixed pulse width (150 µs) and a fixed frequency (24 Hz). This search was performed once a day during 3 days the first and second week. The refinement was then studied with STR then TTR with this subgroup to further obtain stronger contractions of the 3 targeted muscles while limiting contractions of **PT** (Supplementary Material “Electrode’s configuration” section). The same approach was used for the radial nerve. Then, the configurations used were fixed and only the intensity was adjusted if necessary.

This semi-empirical search, based on the actual outcomes of the stimulation, finalized the selection of the configurations and their associated current that induced the best functional key grip or palmar grip with the highest strength and the lowest wrist flexion/pronation which was then assessed. Concerning each patient, the selected configurations that generated the desired functional movement were the following:

- *Participant P1* for the median nerve TLR1 induced palmar grip without **PT** and a weak **FCR**. TLR7 induced a key grip without **FCR** and a weak **PT**. For the radial nerve, TLR2 induced all muscles’ contractions for a full opening of the hand with wrist extension.
- *Participant P2* for the median nerve TLR1 induced palmar grip, STR5 induced key grip. For the radial nerve, STR2 induced a full opening of the hand with wrist extension.

### Recruitment curves confirm the relevance of the empirically selected configurations

**Figure 1:**
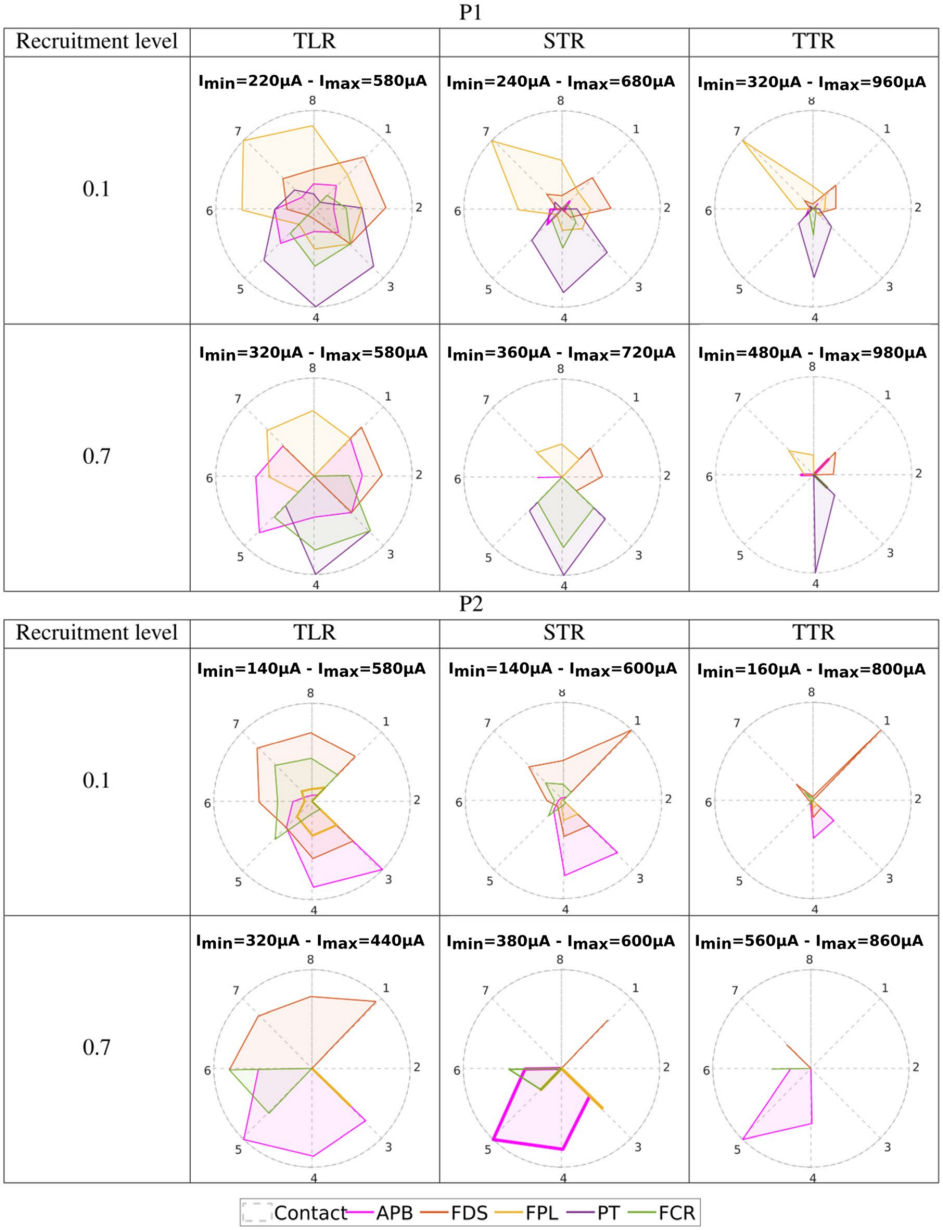

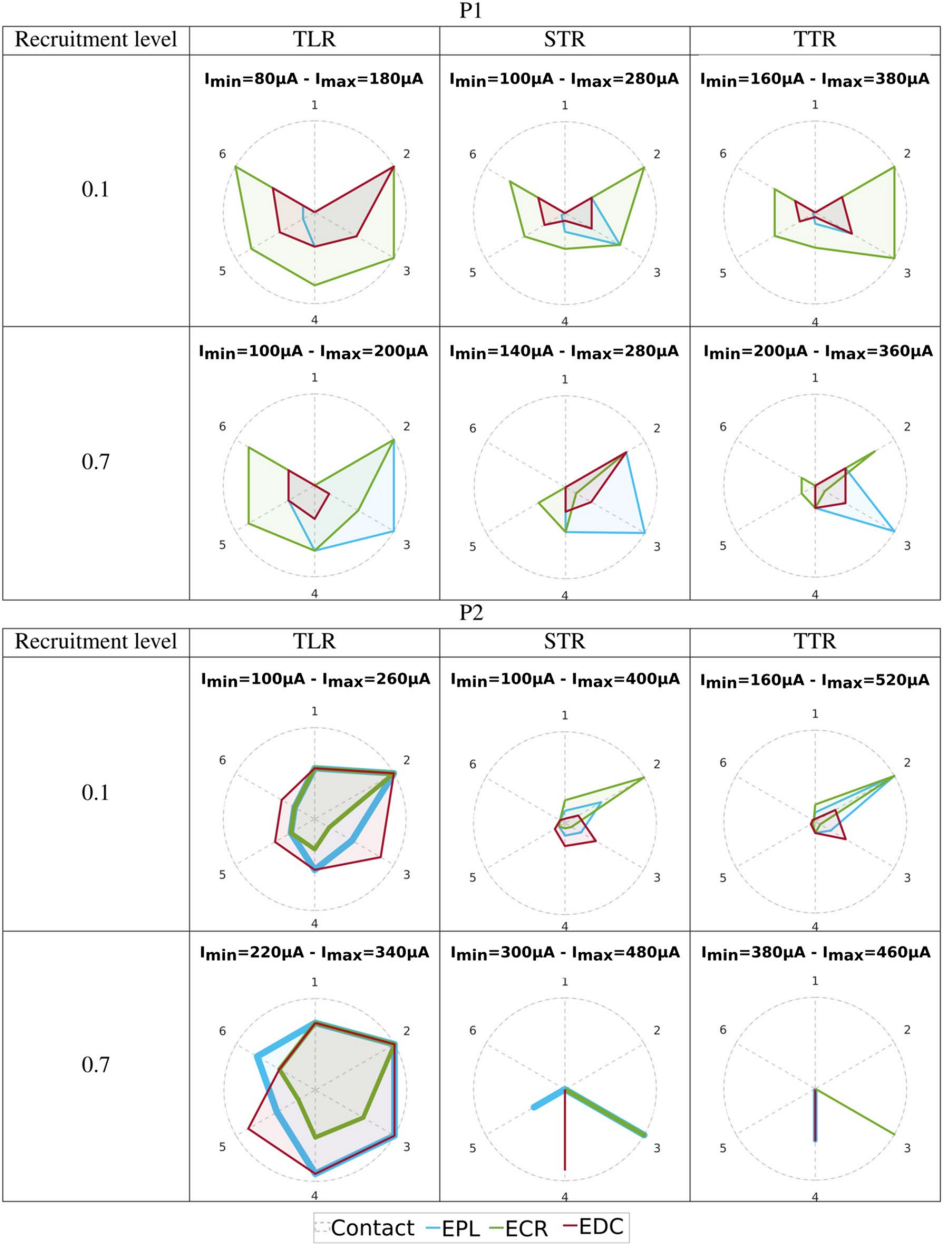

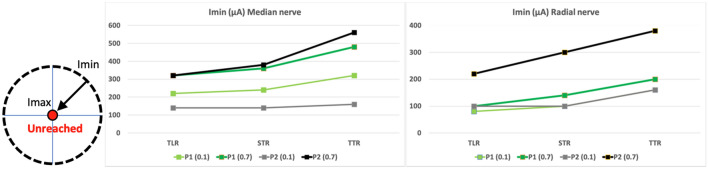
IRO of the muscles for the 3 configurations (TLR, STR, TTR) and for 2 recruitment levels (0.1 and 0.7). From top to bottom: P1, P2 median nerve—P1, P2 radial nerve—Imin values. Each vertex of one colored polygon corresponds to the IRO values for the selected cathode of the considered muscle. The smaller the polygon surface is, the higher the current needed to obtain a recruitment level of 0.1 resp. 0.7 is. A vertex on the circle edge means that Imin, the minimum current amplitude for this configuration, is needed to obtain the given level of recruitment whereas a vertex tied to the center means that the level of recruitment cannot be obtained. In between, along a radius, the sequence of activation with increasing intensities for a given level of recruitment can be seen from edge to center.

Based on the detailed normalized recruitment curves obtained during the last week of the participants' follow up, we computed the Index of Recruitment Order (IRO, see “Methods” section) representing the recruitment order among monitored muscles combined with the amplitude of the intensity needed to reach a given threshold for each (0.1 and 0.7)^34,37^. This index is relative to the electrode configuration and the targeted threshold leading to 12 figures per patient (Fig. 1). For each configuration (TLR, STR, TTR), the IRO varies from 0 (threshold not reached) to 1 (threshold reached with the lowest current’s intensity). It was computed for median and radial nerve responses.

IROs diagram gives information, for a considered electrode configuration, about the selectivity and the synergic sequence of activation of the different muscles. The level of recruitment of 0.1 targets a weak contraction whereas the level of recruitment of 0.7 targets a strong functional contraction^37^. Results exhibit the following statements:

- TLR => STR => TTR differences: diagrams confirm that the selectivity increases from TLR to TTR (less overlapping of polygons). Moreover, as demonstrated in simulations, intensities (Imin) are higher with TTR (Supplementary Fig. 4, Fig. 1). Within a selected cathode, the increment of intensity to activate an additional muscle increases meaning that TTR (resp. TLR) gives the highest (resp. the lowest) discrimination between muscle’s activations (Supplementary Fig. 7). It is due to a smaller extension of the activated nerve’s area in deeper regions when the intensity increases when using more selective configurations^36^.
- First activated muscle: a selective stimulation of a subgroup of muscles is possible with a consistent distribution over the contacts. For instance, for a 0.7 level of recruitment and TLR, a given muscle is predominantly activated by a set of adjacent contacts:
  - For the median nerve, for P1, **FDS** (contacts 1–2) **PT** (contact 4), **APB** (contacts 5–6) **FPL** (contacts 7–8)
  - For the radial nerve for P1 **EPL** (contact 3), **ECR** contacts (5–6)
  - For the median nerve for P2 **FDS** (contacts 7–8-1), **APB** (contacts 3–4-5)
  - For the radial nerve for P2 **EDC** (contacts 5) **EPL** (contacts 6)

For the median, it allows to select different sequences of flexions and thus types of grasping while limiting unwanted movements such as wrist flexion and pronation. For the radial nerve, results are less selective even though it is interesting to note that for P1 a pure extension of the wrist could be obtained using contact 5 or 6. However, the sequences of activation of subsequent muscles differ and allow for different types of opening without the need of accurate individual muscle’s activations. The sequences of activations can be seen in the Fig. 1 for a given contact in a given configuration from the periphery (the first activated muscle) to the center (the last activated muscle). Comparing with the results obtained empirically for the median nerve, we can have a detailed sequences:

- Participant P1: TLR1 (recruitment order **FDS** > **FPL** > **APB** > **FCR** > **PT** at 0.1 **FDS** > **FPL** at 0.7). TLR7 (recruitment order **FPL** > **FDS** > **PT** > **APB** > **FCR** at 0.1 **FPL** > **APB** at 0.7), further confirming weak PT contraction in both cases and at low and high recruitment levels.
- Participant P2: TLR1 (recruitment order **FDS** > **FCR** > **FPL** > **APB** at 0.1 **FDS** at 0.7), STR5 (recruitment order **FCR** > **APB** > **FDS** > **FPL** at 0.1 **APB** > **FCR** > **FPL** at 0.7).

Thereby, the cathode selection allows choosing the order of the sequence of activated muscles linked to the proximity between the selected cathode and the muscles’ group corresponding fascicle (Supplementary Figs. 5, 7). Figure 1 (Imin values) shows that participant P1, for both nerves and all configurations, has smaller Imin values variations to reach 0.1 and then 0.7 than participant P2. Besides, for a targeted level of recruitment (0.1 or 0.7) Imin values are consistently associated to the same pair of inner contact-muscle (except two TLR IROs for P2, Fig. 1): P1-median (0.1 => 7/**FPL**, 0.7 => 4/**PT**), P2-median (0.1 => 1/**FDS**, 0.7 => 5/**APB**), P1-radial (0.1 => 2/**ECR**, 0.7 => 3/**EPL**), P2-radial (0.1 => 2/**ECR**, 0.7 => 3/**ECR**). Finally, Imin values are lower for radial nerve.

Table 1 shows that the level of individual muscle contractions strongly depends on the patient and on the neural selectivity. Indeed, to get an efficient and functional movement, the biomechanical conditions (muscle strength, joint stiffness, rest position) lead to very different stimulation tunings that cannot be a priori set upon general hand biomechanics considerations.

**Table 1:** Normalized recruitment levels of the muscles for the 3 electrode configurations and current intensities selected to evoke the 3 functional movements.

	Radial nerve			Median nerve									
	Hand opening			Palmar grip					Key grip				
P1	TLR2, 80 µA			TLR1, 460 µA					TLR7, 500 µA				
	EPL	ECR	EDC	APB	FDS	FPL	PT	FCR	APB	FDS	FPL	PT	FCR
	0.13	0.62	0.14	0.6	0.72	0.69	0.08	0.11	0.09	0.36	0.84	0.11	0.04
P2	STR2, 240 µA			TLR1, 360 µA					STR5, 440 µA				
	EPL	ECR	EDC	APB	FDS	FPL	PT	FCR	APB	FDS	FPL	PT	FCR
	0.48	0.32	0.3	0.1	0.9	0.11	–	0.33	0.81	0.53	0.6	–	0.56

The common stimulation parameters are: frequency 24 Hz and pulse width 150 µs.

**Figure 2:**
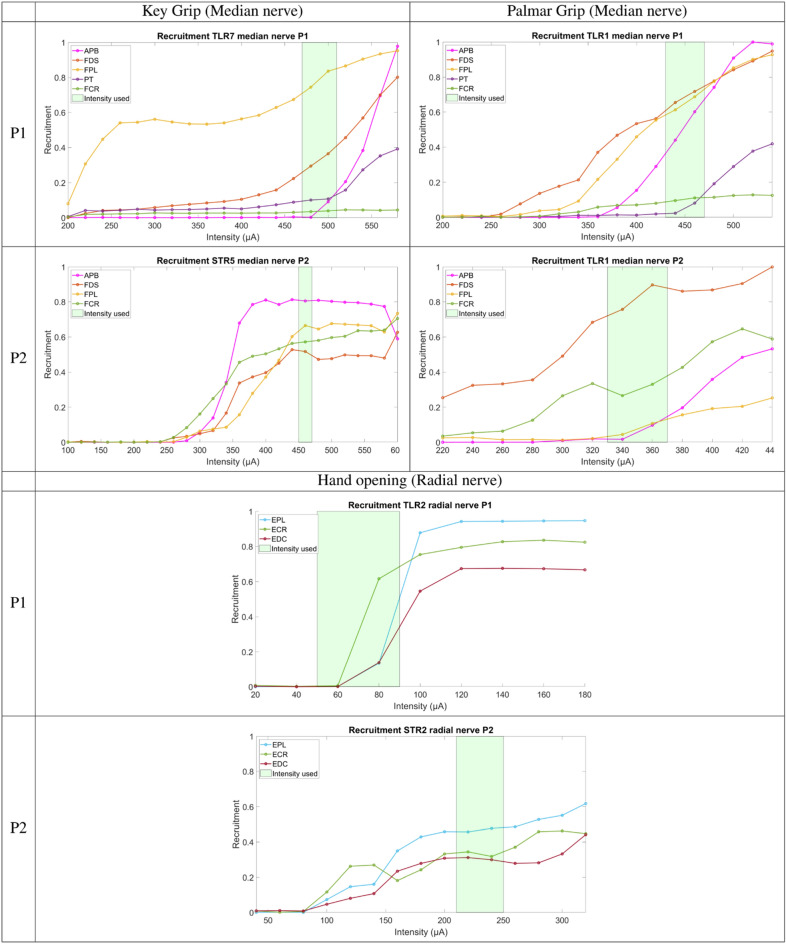
Recruitment curves of the 3 selected configurations evoking functional movements: Participant P1: TLR2 for hand opening (radial nerve), TLR1 for palmar grip, TLR7 for key grip (median nerve). Participant P2: STR2 for hand opening (radial nerve), TLR1 for palmar grip, STR5 for key grip (median nerve). The green areas show the ranges of usable intensity settings that allow modulating the force while keeping a similar muscles’ synergy.

The recruitment curves presented in Fig. 2 lead to several interesting observations. The recruitment order is depending on the intensity so the sequential recruitment based on an arbitrary threshold (0.1) is only indicative. For instance, for P2 on the median nerve, the relative levels of the recruitment between muscles are changing while the intensity is increasing leading to a different recruitment order: for STR5, **FCR** is the first recruited muscle but **APB** raises the highest plateau (0.81). It means that an objective selection of the configurations and the contacts based on recruitment curves only is almost impossible as a unique relationship between a desired outcome and a configuration/contact/recruitment level cannot be established.

### Functional assessments of the selected movements

As explained above, the recruitment curves are not sufficient to describe the functional outcomes. Indeed, the gripping function only makes sense in relation to the manipulated object. It is the interaction between the hand and the object that makes it possible to objectify the grasping function. Thus, the extension of the fingers and the wrist must be adapted to the volume of the object to be grasped and the flexion of the fingers and the wrist must be adapted to the object volume and weight to allow grasping and moving it.

**Figure 3:**
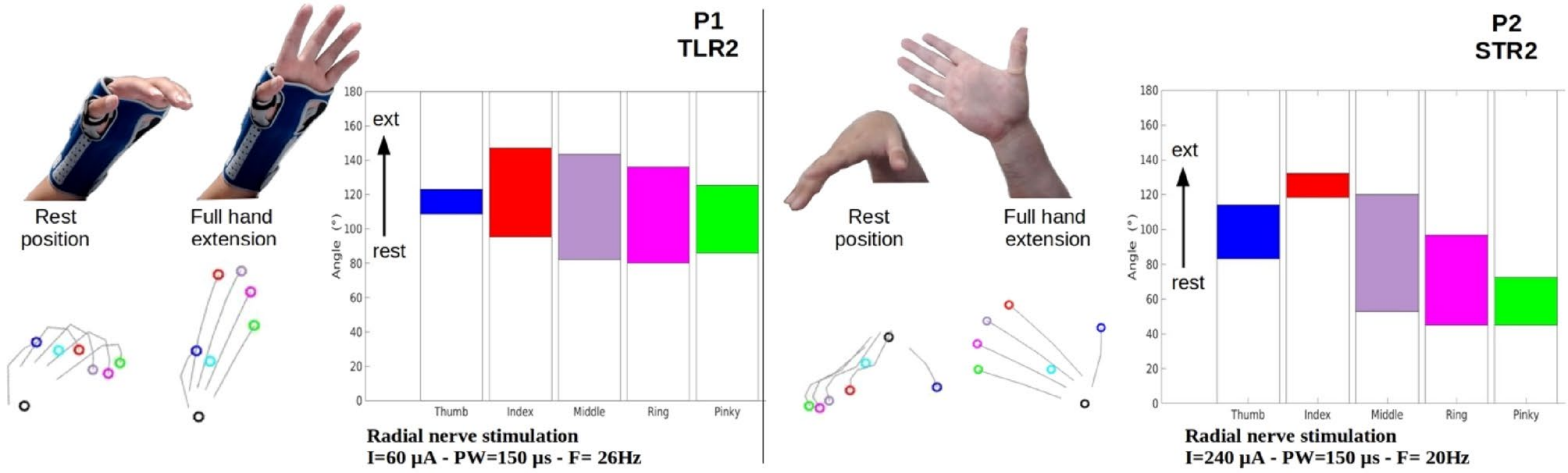
Kinematics data of hand opening. Left: participant P1—Radial nerve stimulation TLR2 (Wrist brace + Thumb splint). Right: participant P2—Radial nerve stimulation STR2 (Thumb splint). Video snapshots and posture reconstruction based on Leap Motion data. The diagrams represent the excursions of the 5 angles.

**Figure 4:**
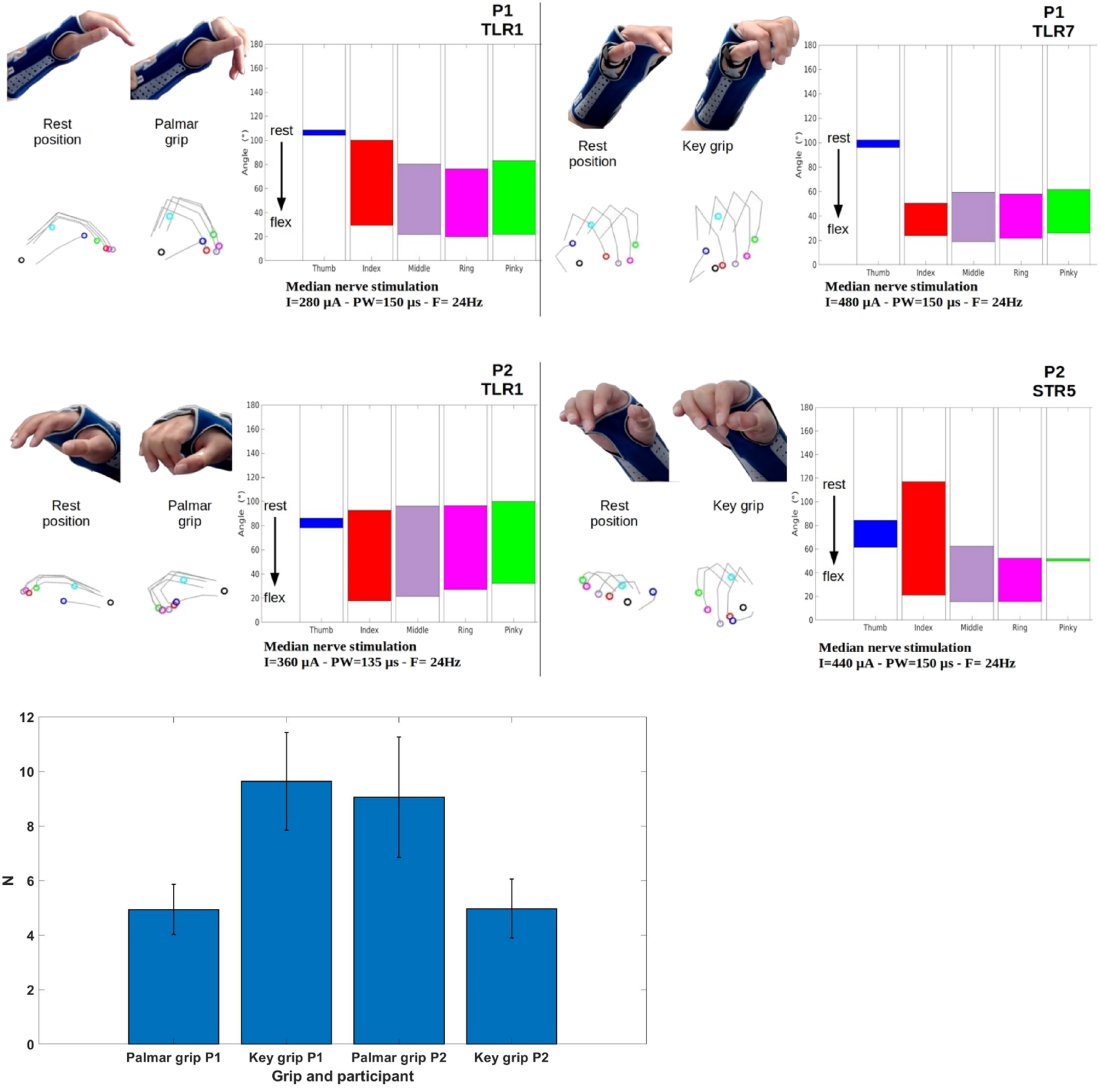
Kinematics data of palmar grip with thumb and key grip. Top: Participant P1 (Left: Configuration TLR1, Right: Configuration TLR7). Middle: Participant P2 (Left: Configuration TLR1, Right: Configuration STR5). Video snapshots and posture reconstruction based on Leap Motion data. The diagrams represent the excursions of the 5 angles. Bottom: Normal forces recorded for Palmar (instrumented can) and Key grip (instrumented tablet) for P1 and P2, 3 trials per condition.

Video recording and kinematic data acquired with the Leap Motion corresponding to the selected stimulation configurations were processed and synthesized in Figs. 3 and 4 to illustrate the obtained movements. The selected configurations and intensities provided an efficient and wide opening of the hand allowing the patient to approach objects before grasping and finally releasing objects. The quality of the opening motion can be assessed by the fact that the fingers and the thumb are extended sufficiently to approach and surround an object such as a can of 70 mm diameter. Depending on the object size, the amplitude of the extension can be adjusted by increasing current intensity. In the chosen examples (Fig. 3), participant P1 was wearing a wrist brace and a thumb splint while participant P2 was only wearing a thumb splint. The wrist brace kept the wrist in an appropriate position, i.e., dorsiflexion, while activating the finger flexors (Fig. 3).

Grasping movements were assessed by the data provided by Leap Motion device and the corresponding videos. We must highlight the importance of the initial posture: depending on the initial joint angles, applying a stimulation pattern leads to a different final posture. We have therefore equipped the participants with a wrist brace to start from a neutral resting posture for the wrist. In Fig. 4, the two main grasping postures obtained with the two participants are described. The so-called palmar grip with the thumb corresponds to a flexion of the fingers with the thumb over the fingers. In the key grip, the pulp of the thumb is applied to the radial edge of the index finger at the second phalanx. In the functional tests with manipulation of an object, the closing of the hand was preceded by an opening of the hand and an object constrained the finger paths.

The quality of the grasping is difficult to predict without the interaction with objects. Therefore, the assessment was further completed with instrumented objects allowing to estimate the contact forces exerted by the fingers. A bar (similar in thickness to a chocolate bar) was instrumented for the key grip and a can (similar in size to a soda can) for the palmar grip (see “Method” section). The forces induced by the stimulation were sufficient to maintain the object firmly over time. Forces are computed over 3 averaged trials.

The Table 2 shows that the kinematics without object is very difficult to interpret as similar positions are obtained for a given patient except the pinky not supposed to be activated (but mechanically constrained) and the thumb that shows a larger flexion for key grip over palmar grip. Concerning recruitment level, **FDS** recruitment is higher for palmar grip whereas **FPL** recruitment is higher for key grip. **APB** recruitment seems counterproductive concerning the produced force and not linked to the obtained grasping.

**Table 2:** Combined assessment of grasping movements.

	Muscle recruitments APB-FDS-FPL	Force	Kinematics (final angle) Thumb-Index-Middle-Ring-Pinky
P1 Key Grip	0.09 0.36 **0.84**	9.6 N (± 1.8 N)	**96°** 24° 19° 22° 26°
Palmar Grip	**0.6 0.72** 0.69	4.9 N (± 0.9 N)	105° 29° 22° 20° 22°
P2 Key Grip	**0.81** 0.53 **0.6**	4.9 N (± 1.1 N)	**61°** 21° 15° 16° 50°
Palmar Grip	0.1 **0.9** 0.11	9 N (± 2.2 N)	78° 18° 22° 27° 32°

Significant values are in bold.

**Figure 5:**
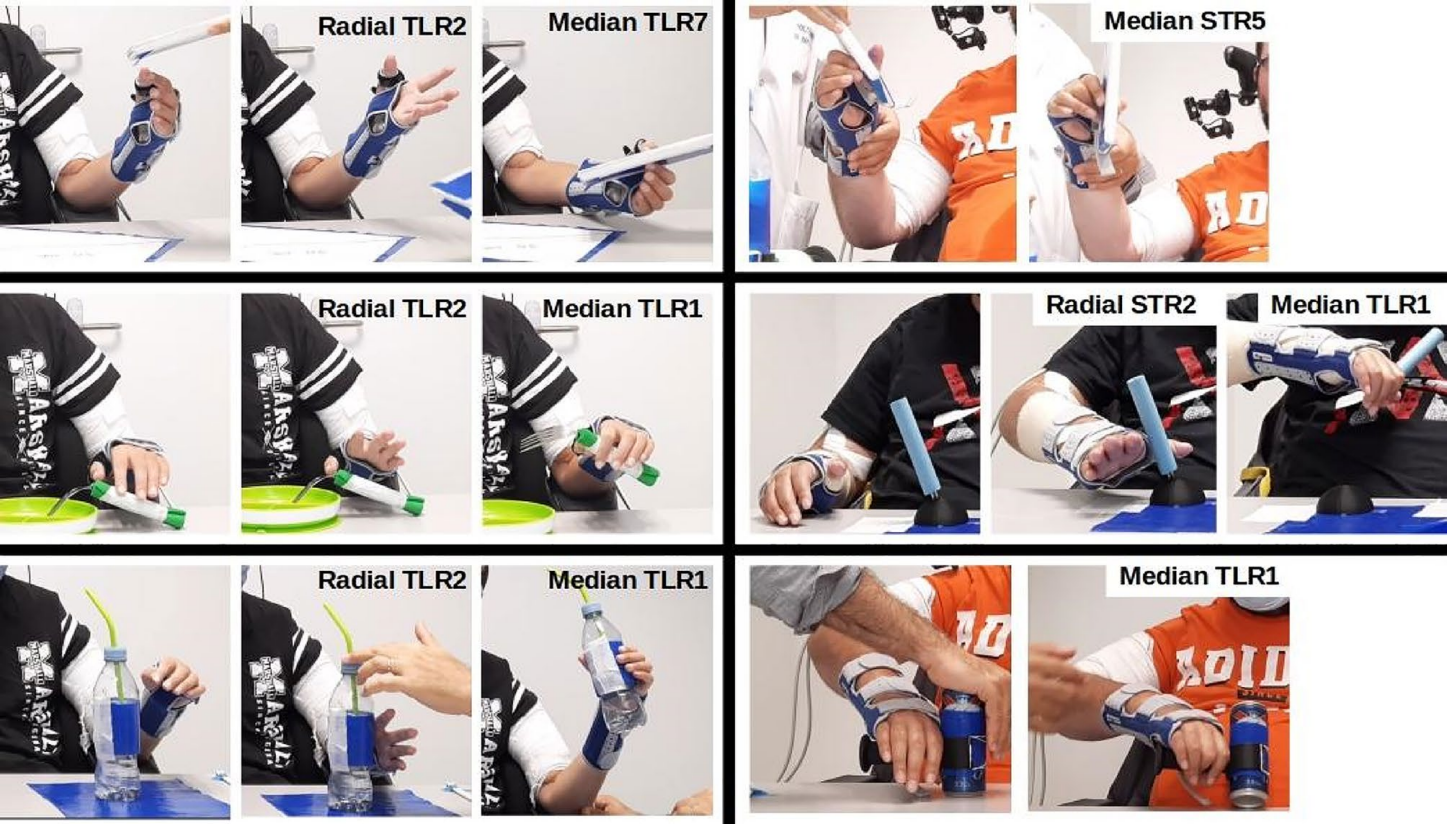
Video snapshots illustrating different grasping performances. Left: participant P1. Right: participant P2.

Over the 28-days trial a large number of grasping tasks have been achieved with different objects to assess functional outcomes. In Fig. 5 we have reported the most representative tasks that were realized i.e. chocolate bar (250 g) pick and place, fork with food intake, pen with lines drawing, half a liter bottle (500 g) manipulation and drinking with a straw, 330 ml can (330 g) manipulation. To control the triggering of the 3 pre-programmed stimulation configurations: (1) hand opening (object approaching or releasing), (2) digito palmar grip with thumb, (3) key grip, according to the state machine described (“Material and Methods” section), participant P1 used the voluntary contraction of the platysma and upper trapezius muscles, participant P2 used two occipital buttons.

## Discussion

### Variability of the settings

The results show that the inter individual settings are completely different even though the ranges of the values remain in the same order of magnitude. It means that the technology can have generic specifications but needs to be personalized: the selected contact, the configuration, and the used intensity ranges reflect this variability. The obtained movements are also quite different (wrist position, force), due to participant biomechanical conditions that are drastically different, but the functional outcome is similar showing that functional tasks are the final, thus important assessments to be considered.

On the contrary, intra-individual variabilities are extraordinarily low, further confirming the interest of implanted technology. The optimal configurations (TLR vs. STR vs. TTR) did not change during the first phase of blind evaluation and stimulation settings remained identical over time: the frequency, the pulse width were never changed, and the intensity slightly adjusted no more than one step up or down (± 20 µA). This means that the settings remained the same from session to session, showing stable muscles’ response and selectivity. These results are sustained by highly stable impedance measurements and thresholds over time (Supplementary Figs. 3, 4). We obtained very low current intensity thresholds (median nerve P1 260 µA (± 62 µA), P2 184 µA (± 33 µA) and radial nerve P1 80 µA (± 0 µA), P2 100 µA (± 20 µA)) for muscle activation (the lowest thresholds obtained for each muscles over all the configurations). The values are similar to the thresholds reported in the literature^25,27^ but lower than those obtained in our own previous clinical trial^35^. In addition, we observed only small variations in these thresholds over the 28 days of follow up (no more than one step of current i.e. 20 µA). Moreover, the settings for the 6 functional configurations described in Figs. 3 and 4 were followed up and showed stabilized tuning during the last week of the clinical trial (Supplementary Fig. 5). We had frozen the stimulator characteristics to conform to the state-of-the-art reported values which forced us to use a too high intensity step (20 µA) preventing us from getting more accurate settings and smoother recruitment curves. This was partially compensated by pulse width modulation, but a higher resolution would benefit future tests. While removing electrodes, no fibrosis was detected between the electrode contacts and the nerve tissues^38^, the self-adapting epineural electrode gently and intimately surrounding the nerve. A thin fibrotic tissue encapsulated the whole electrode increasing the mechanical adherence to the nerve without stressing the tissues. It may explain these high stability.

### Contributions and limitations of the proposed approach

The first contribution is that we succeeded, for the first time, in repeatedly generating 3 functional movements of the hand during the 28 days of implantation with solely 2 epineural multicontact cuff electrodes. We validated the concept that we had primarily studied through simulations and theoretical optimization followed by an original design of both the electrodes and the stimulator. Indeed, two multi-contact epineural electrode cuffs were implanted around the radial and median nerves of two participants with complete C4 spinal lesion. The electrodes were in place for 28 days during which the participants were involved in various sessions to tune the stimulation configuration parameters, to adjust the piloting interface and to perform functional tests^38^. Both participants were able to trigger 3 movements using their own voluntary actions (activating muscle contractions or occipital buttons)^38^: hand opening, key grip and palmar grip. Different objects were seized and handled by the participants. The produced torque for both grasps is high enough (> 4 N) so that the majority of daily activities can be safely performed^12^.

The second important contribution to the generation of movements was to show, through the search for muscle synergies, i.e. the activation of several muscles at different levels with a single pulse, a very efficient and new way of tuning such a neuroprothesis compared to classical tuning muscle by muscle. Indeed, scanning configurations is equivalent to looking for the synergic movements search as median, resp. radial nerve, innervate essentially synergic muscles. Searching for highly selective, individual muscle activation that should be further combined to provide functional movements appeared to be much more complex and less efficient.

However some limitations also appeared. First, surface EMG is known to include crosstalk between muscles. Our method allows to extract separate M-waves (see Supplementary Material and William et al*.*^39^) but needs to be further confirmed; however the matching between sorted M-waves and individual muscle contraction was assessed by the consistency between recruitment, EMG electrode’s location over targeted muscles and visual inspection of induced movements. Wired EMG, or High Density EMG as a non-invasive method, could be used to consolidate our approach in a future work^40^. Concerning recruitment curves, it should be further confirmed that they are stable over time. We recorded them only once at the end of the protocol to assess the link between the chosen configurations and the recruitments, but the stability of the settings were checked only through thresholds, impedances and intensity settings (see Supplementary Material).

A second limitation concerns the obtained movements that were insufficient to provide stable grasping with an object without using a wrist brace. This is because an extension of the wrist while performing the grasping function is necessary to secure and provide a reliable functional movement. The wrist position drastically changes the resulting torques generated by a constant stimulation current. This is due to the complex biomechanics of the hand together with the properties of the muscles, in particular the force–length relationship. In addition, as in all other approaches based on FES to restore grasping, we used open loop stimulation which makes the tuning to obtain an effective grasping very challenging as it depends on hand-wrist posture. We solved these problems with a wrist brace to allow for a neutral resting posture of the hand which facilitates stimulation parameters tuning. Thus, the orthosis blocks the wrist flexion so that the fingers and thumb flexions are reliable and more importantly, reproducible. In the absence of the brace, the fingers’ flexion can induce wrist flexion that further decreases the efficacy of the grasp. However, our approach allows for a combination of a pure wrist extension via selective radial nerve stimulation together with median nerve stimulation in order to stabilize the wrist by co-contraction without the need for a splint. Preliminary tests in this way with P1 were encouraging with a successful co-contraction of wrist extension and fingers flexion (Supplementary Material “Advanced posture management with co-contraction” section). Nevertheless, in this study, we decided to focus on the reproducibility of evoked movements over 28 days rather than exploring new stimulation combinations: the developed device achieved the goal of providing an autonomous hand that opens, grasps and releases everyday objects in less than 3 weeks of adjustments, adaptations and rehabilitation.

**Figure 6:**
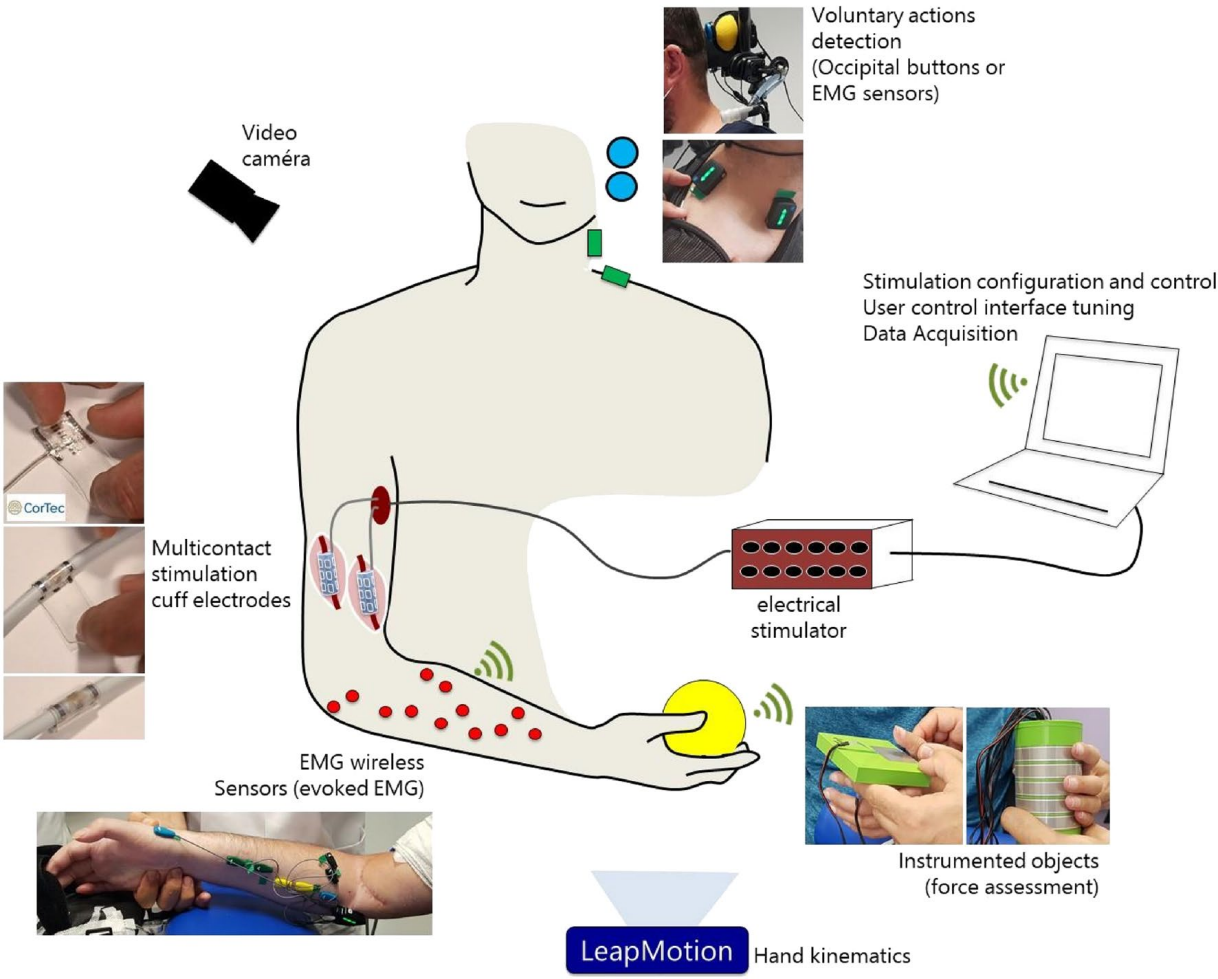
Setup description. An experimental platform was developed to control the stimulation delivered to 2 neural epineural electrodes implanted around the median and radial nerves. Evoked electromyography, video, evoked movement kinematics and grasping forces were recorded. The participants used voluntary muscle contractions or occipital buttons to trigger different stimulation configurations.

Another important lesson learned in this study concerns pronation-supination movements. We did not attempt to control these movements, which were considered undesirable. In this case, selectivity is used to avoid **PT** activation. These movements depend not only on muscle activation but also on wrist joint stiffness. This was specifically the case for participant P2. Despite our attempts to control the pronation by means of braces, in the end we decided to adapt the objects to allow an approach and grasping of can-type objects (Fig. 6). This is a usual practice of occupational therapists who adapt everyday objects to the motor abilities of patients. In this case, we have succeeded in proposing a functional grasp by means of an accessory (3D printed handle) allowing a grasp at 90°. The solution is simple and effective and raises the question of the balance between the use of complex stimulation paradigms and the use of adaptive tools or even light passive orthoses. Of course, the grasping/releasing of objects itself remains under the exclusive control of the FES since it is an active movement.

Another limitation of this protocol is that all-or-nothing stimulation paradigms without the ability to modulate the stimulation during execution was used. For the opening of the hand, it does not seem necessary to go further in the complexity for the approach or the release of objects. In contrast, for grasping, a progressive closure of the fingers around the object could help the patient to obtain a more reliable grip by avoiding the use of an immediate strong contraction which could lead to an incorrect positioning of the fingers around the object.

### Guided tuning procedures

Tuning stimulation parameters procedures were based on a mixed approach. The simulations studies gave a reduced set of relevant selective configurations (TLR, STR, TTR) that allows to study selectivity. The first systematic scan of all inner contacts (as cathode), with and without holding an object, was then possible in a limited time as only the intensity needed to be adjusted. Assessment of the selectivity (which muscle is activated alone over which range) and synergies were then very simplified. Among the subset of inner contacts using TLR configurations that provide functional movements, the guided search continued through testing more selective configurations (STR then TTR). For instance, we succeeded to increase the selectivity in such a way that undesired movement, i.e. wrist flexion or pronation, was further limited while keeping the desired synergic activations. It finally showed that highly selective configurations were not the best ones (TTR) confirming that synergies (obtained with TLR eventually STR) are better than isolated then combined muscle’s activation. This is a strong advantage of our approach versus epimysial/intramuscular stimulation for which synergies must be found through multiple muscle’s activations and so current settings. Eventually, the fact that obtained synergies differ depending on the inner contact used confirms that a functional fascicularisation exists in the human's upper limb, as previously suggested^41–43^, and can be exploited.

However, our guided approach limitation concerns the use of the recruitment curves. These were long but necessary sessions to assess the recruitment logic i.e. a progressive and selective activation of muscles’ groups with a similar recruitment order on a specific inner contact whatever the configuration is (TLR, STR, TTR). The more selective configurations allowed smoother transitions, more isolated muscle’s contractions, and slightly different recruitment orders to possibly increase the accuracy of the tuning, but a direct link between recruitment curves and functional outcome is still not obtained so these curves cannot be used as a first step of tuning. The concept of selective stimulation and tuning should be revisited in a clinical context to limit the duration of sessions and to get closer to an objective and quantified tuning. These curves can rather be used to finalize the tuning through the fine tuning of the intensity or possibly to switch from a configuration to another that have a similar recruitment order (same synergy) but not the same recruitment levels. We did not go into this step as it would have need further sessions. To do so, both the scanning procedures and the assessment tools should be improved. Clearly, an efficient grasp cannot be obtained automatically. The fact that the hand biomechanics complexity, the shape and the weight of the object to grasp all have a strong influence on the quality of the grasping makes it impossible to make predictions from recruitment curves, or even from a complete movement without any objects. A tool to quantify objectively the grasping while scanning the configuration is paramount. There is no solution to date and it will be considered as a central topic for the next trials.

As a whole, the minimally invasive approach we propose is well fitted to clinical transfer as the surgery is limited compared to epimysial approaches, very stable and energy efficient and thus easy to use from day to day compared to external stimulation approaches with an efficient guided empirical search.

Further improvements concern the elbow flexion extension that could be addressed by either a more proximal radial stimulation or the musculocutaneous nerve stimulation. It would extend the eligible group of patients with still at most 3 neural cuff electrodes. However the selectivity challenge would be greater to obtain pure elbow movements and should be proved. Besides, the control by the patient is different in nature as it concerns the object’s approach and not the gripping itself. Combined approaches with functional surgery may be also a solution, in particular for elbow flexion recovery^8^. Further researches are necessary to keep the solution simple with hidden complexity of the technology and richer interfaces^44,45^.

## Conclusion

This clinical trial is a proof of concept of the ability of the selective neural stimulation to provide synergic and functional hand movements. It further confirms, for the first time, that with only 2 epineural electrodes the essential hand movements, i.e. opening, key grip and palmar grip, can be obtained with reliable and reproducible stimulation settings. Finally, contrary to most of the previous approaches, we further demonstrate that a synergic muscle activation is easier to set compared to the individual setting of each muscle contribution. Rather than using selectivity to isolate each muscle’s contraction, it allows to select a set of muscle synergies.

## Materials and methods

### Subject recruitment and surgery

Two male participants with a traumatic SCI C4 AIS A were included in the study (Supplementary Table 1). Participants provided written informed consent before participating in accordance with the Declaration of Helsinki. The protocol was approved by the French Ethics Committee (CPP Ouest IV Nantes, France, ID-RCB #2019-A00808-49) and French Health Agency (ANSM). The study was registered on ClinicalTrials.gov (registration number: NCT04306328 first registered 12/03/2020). Patients gave informed consent to publish photographs and videos acquired during the protocol and included in the present paper*.*

The participants underwent a first surgical procedure to implant the median and radial nerves epineural electrodes located above the elbow. After 28 days the electrodes were explanted during a second surgical intervention. During 28 days the participants were hospitalized and underwent 3 weekly trials for the adjustment of stimulation patterns, as well as daily rehabilitation sessions and clinical tests. The 28-day duration is below the 30-days limit that allows a clinical trial to be legally classified as a short term trial (Online Annex IX, Section 1, EU directive 93/42). A long-term trial will be the next step with an implanted stimulator and thus without percutaneous wires. Detailed surgical procedures and clinical scores are presented in Azevedo et al.^38^.

Figure 6 presents the setup used to explore and evaluate the functional movements obtained with all the tested configurations on both nerves. Next sections detail the different parts of this setup.

### Electrodes

2 electrode cuffs were used, both composed of 2 external rings and a number of inner contacts that depends on the targeted nerve: (i) 3–4.5 mm diameter (self-adjusting), 2 cm length epineural electrode was used for the radial nerve (6 inner contacts, Cortec Gmbh, Freiburg, Germany) and a 4.5–6.75 mm diameter (self-adjusting), 2 cm length epineural electrode was used for the median nerve (9 inner contacts, Cortec GmbH, Freiburg, Germany). The epineural electrode inner contacts sizes are 2.4 × 0.8 mm^2^, 2.4 mm spacing between two adjacent contacts (center to center) and made of 90/10 Pt/Ir alloy embedded with silicone. The distance between each external ring and each inner contact is 0.8 mm (center to center).

Electrode integrity was checked all along the 28-days by an impedance measurement before each working session i.e. 12 times. The impedance was estimated by the ratio between the voltage and the current at the end of the cathodic phase of a bipolar balanced stimulation between each contact and the proximal ring. The stimulation parameters were set to 60 µA, 300 µs, 4 Hz, 5 pulses. The first pulse was discarded and the 4 remaining were averaged.

### Stimulation protocol

The stimulator architecture is described in^46^. It can distribute the current over the 9 inner contacts on the median (respectively 6 on the radial) and the 2 rings of each electrode with a ratio between 1/15 and 15/15 of the total injected current. This makes it possible to drive independently the amplitude of multiple current sources in synchrony for each of the 8 or 11 contacts of each epineural electrode: this provides a unique and innovative way of shaping the current in 3D within the cuff electrode. Each contact can be further configured as anode or cathode during the active phase of the stimulus. The current intensity (up to 5 mA, 8-bit resolution), pulse width (up to 510 µs, step 2 µs) and frequency (up to 50 Hz) are configurable and the compliance voltage is 20 V. The stimulator follows the essential safety requirements concerning both the embedded software and the hardware. The stimulator was fully insulated from the control PC. The waveform stimulation was biphasic, symmetric and charge balanced with a delay of 100 µs between the active phase and the recovery phase^47^. To evaluate the selectivity of the multicontact electrode, we selected up to 3 configurations that we compared with the conventional bipolar ring configuration (Supplementary Table 2) based on a previous simulation study and validated in preclinical studies^34,36^. The stimulation scanning is initiated with the threshold value that induces a visible contraction with the Ring configuration. Then, an automatic scanning with increased steps of 20 µA is performed until obtaining a plateau (EMG recordings) or a contraction that is too strong, at which point the procedure was stopped upon the medical doctor request. The configurations and intensities were increased every 1 s (0.5 s ON–0.5 s OFF) to limit fatigue.

The pulse width and frequency were fixed to 24 Hz and 150 µs. With 24 Hz we checked that no muscle tremor was induced.

### Patient control

3 command modalities have been proposed to participants to control the triggering of hand opening and 2 different grasping: (1) they could perform different movements with their contralateral shoulder that were captured with inertial sensors (IMU)^44^; (2) they could use two different muscles voluntary contractions, again from the contralateral side, that were captured by electromyography (EMG) sensors; or (3) they could push buttons attached to the wheelchair headrest with head movements. P1 chose to use small voluntary contractions of the supralesional platysma and upper trapezius muscles (of the contralateral side of the stimulated hand) detected by surface EMG (Trigno™ Delsys, Natick, MA). EMG was rectified and low pass filtered (6 Hz low-pass Butterworth, 4th order) to extract the envelope and a threshold was set for each EMG sensor on each muscle so that P1 could clearly activate the command when desired, but not by accident when speaking or laughing for instance. P2 used the head command (2 push buttons) as he was not able to contract his muscles in a reliable manner to allow using EMG detection modality. Besides, controlling contralateral movements, although possible, was inducing rapid fatigue onset leading the participant not selecting IMU modality.

A finite state machine (FSM) was defined to associate the user’s commands (detection of EMG threshold transitions or occipital buttons’ pressing) to actions depending on the current FSM state. The actions were associated with predefined stimulation configurations. Once the system was turned on, the first user command received was always decoded into triggering “open hand” stimulation configuration. Then, the participants could choose one of the two preset grasping (key grip or palmar grip). The next action was always “open hand” again, regardless of the command received. Finally the next command, whichever it was, would deactivate the stimulation. The FSM was customizable so that each participant could choose which command would trigger which stimulation action.

### Evoked-EMG processing

The muscle response to electrostimulation was characterized by recruitment curves obtained from EMG recordings. Surface EMG was preferred to intramuscular EMG to limit the risk of infection and bruises. Moreover, the higher selectivity of intramuscular electrodes was mitigated by the fact that neural stimulation induces M-waves on a limited and known subset of muscles. Besides, we developed a robust post processing able to rebuild individual M-waves^39^. EMG were recorded with a sampling frequency of 2222 Hz (Quattro™ Delsys, Natick, MA). EMG data are then filtered to eliminate residual DC (High pass, order 1, Cutoff frequency 1.5 Hz) and high frequency noise (Low pass Butterworth, order 4, Cutoff frequency 400 Hz). EMG are synchronized with the stimulator so that for each stimulus resulting compound evoked EMG are recorded and then averaged for each intensity step. The period is 42 ms with a 500 ms Onset, therefore about 13 evoked-EMG responses are averaged for each intensity level. Even though each EMG sensor was targeting a single muscle, almost all EMG channels were capturing more than one muscle due to the proximity of each other (Supplementary Fig. 9). To separate the different muscles’ contributions we used crosstalk cancellation when a channel provides a single M-wave followed by Meyer wavelet analysis because of its bounded frequency content, to extract each component through the determination of their specific, non-overlapping time–frequency expansion^39^. The recruitment curves are computed using the RMS value of this time–frequency area for each identified muscle (see Supplementary Material “Recruitment curve’s details” section).

Sufficiently non-overlapping time–frequency areas for **ECR, EPL, EDC** of participant P1 (radial nerve stimulation) was not identified to avoid crosstalk. Probably due to the fact that the patient had a thin forearm, muscles were very close to each other. M-waves could be recorded on all channels but with similar time–frequency components. In this complex case we developed an original method, out of the scope of the present paper. In brief, our approach consists in searching for the mixture of up to 3 parametrized synthetic Action Potentials modeled by piecewise gaussian-like curves. This powerful method leads to the clean separation of M-waves but with a more demanding computation time compared to wavelet analysis and a quite complex parametrization of the synthetic Action Potentials.

The recruitment curves were then normalized to the maximum evoked-EMG for each muscle and each patient over the whole session (all configurations, all intensities). For patient P2 the **PT** EMG for the following configurations: Ring, TLR1, TLR3, TLR4, TLR5 and TLR6 data was corrupted and therefore the PT tracking was discarded for P2.

Eventually, so-called Index of Recruitment Order (IRO) is computed as follows: for a given configuration (TLR, STR or TTR), and a given recruitment level (0.1 or 0.7):

- For each muscle *‘m’* and for a given inner contact *‘c’* the intensity $I_{m,c}$ needed to reach the given level of recruitment is determined.
- For a given inner contact, muscle’s responses are ranked and weighted $W_{m,c}$. This value decreases linearly from 1 (for the muscle that reaches the level of recruitment first) to 0 if the muscle does not reach the level of recruitment). For instance if 3 muscles for a given contact reach the level of recruitment among 5 muscles $W_{m,c} = \left[ {1, 0.8, 0.6, 0, 0} \right]$.
- To normalize the value within a given configuration, $Imi{\text{n}} = \mathop {\min }\limits_{m, c} \left( {I_{m,c} } \right)$.
- $IRO_{m,conf,c}$ for each muscle, each contact, each configuration is then computed as follows: $IRO_{m,c} = W_{m,c} *Imin/I_{m,c}$. A value of 1, for a given configuration, is thus always attributed to the muscle for which the recruitment reaches the given level of recruitment with the lowest intensity whatever the contact.

This computation is then repeated 2 nerves × 3 (configurations) × 2 (levels of recruitment) × 2 (patients) times represented in Fig. 1.

### Evoked movements kinematics analysis

**Figure 7:**
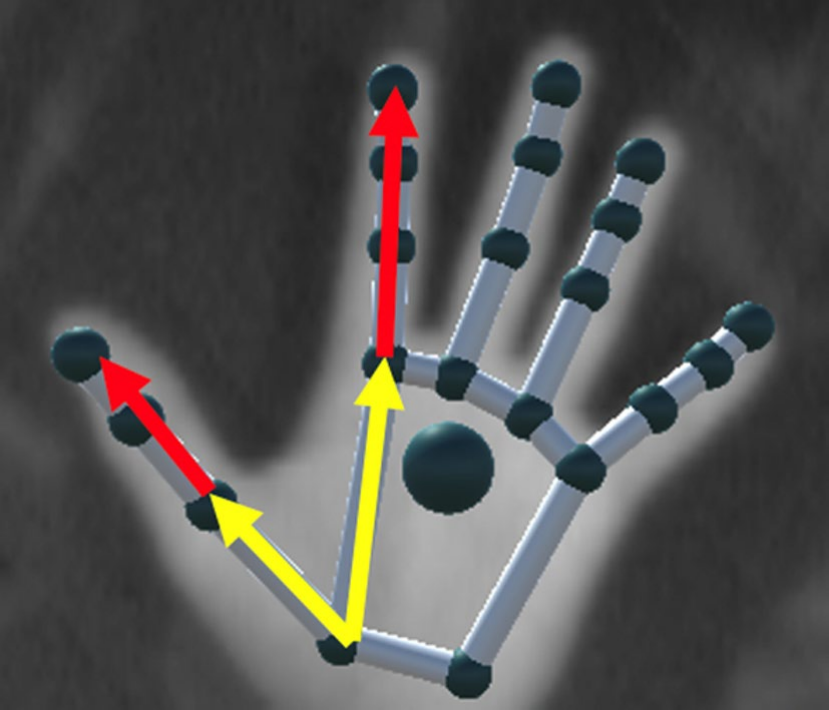
For each finger, the angle between the metacarpal segment (yellow arrow) and the extremity of the last phalanx is computed (red arrow). For the thumb the first phalanx is taken into consideration instead of the metacarpal segment.

A video camera and a Leap Motion Controller (Leap Motion, San Francisco, CA, USA) were synchronized with the stimulator and Delsys system. Leap Motion provides the 3D positions and orientations of the bones and joints of the recorded hand. The data is processed (Fig. 7) using a time window of 1 s before stimulation is ON (to get the average position at rest) and after the stimulation is ON (to get the averaged final position with the desired movement). 180° describes a full extended finger while 0° describes a full flexed one.

### Instrumented Objects

Two objects were 3D printed and equipped with Force Resistive Sensors (FSR, Ohmite Manufacturing, Warrenville, IL, USA) in order to get an estimation of the strength applied by the fingers: (1) a 125 g and 70 mm diameter can equipped with 5 FSR02CE (10 mm strips cut to fit the can) located under the 4 fingers and 1 FSR01CE (40 × 40mm squares) located under the thumb for palmar grip condition and (2) a 55 g tablet of 15 mm height equipped on each side with one FSR01CE for key grip condition. Data was recorded via the Delsys system. The sensors were calibrated with weights (50, 100, 200, 500, 1000 g) before being mounted on the objects. The obtained curves for each sensor type were approximated with second order polynomials (one for FSR01CE and one for FSR02CE). These relationships were used to convert the measurements into forces. The examiner directed the fingers during the movements in order to position them in front of the sensors.

### Clinical trial ethics committee approval, registration, regulation and guidelines

The protocol was approved by the French Ethics Committee (CPP Ouest IV Nantes, France, ID-RCB #2019-A00808-49) and French Health Agency (ANSM). The study was registered on ClinicalTrials.gov (Registration Number: NCT*04306328 first registered 12/03/2020).* It follows Helsinki declaration. The EU Directive 93/42 applied and ISO 14155:2011 (Clinical trial good practice) was followed.

## Supplementary Information

Supplementary Information 1.

Supplementary Video 1.

Supplementary Information 2.

## Data Availability

All data analyzed during this study are included in this published article and its supplementary information files. The raw datasets generated during the current study are not publicly available due to their clinical status and their link to a restricted set of patients but are available from the corresponding author on reasonable request.
