# Supplementary material for: Activating effective functional hand movements in individuals with complete tetraplegia through neural stimulation
Source: Sci Rep. 2022 Oct 6;12:16189. doi: 10.1038/s41598-022-19906-x (PMC9537317; doi:10.1038/s41598-022-19906-x)
Supplement: Supplementary file 1 — Supplementary Information 1. [file 41598_2022_19906_MOESM1_ESM.docx]

**Activating Effective Functional Hand Movements in Individuals with Complete Tetraplegia Through Neural Stimulation**

**Authors:** Christine Azevedo Coste^1,*^, Lucie William^1^, Lucas Fonseca^1^, Arthur Hiairrassary^1,2^, David Andreu^2,3^, Antoine Geffrier^4^, Jacques Teissier^5^, Charles Fattal^1,6^, David Guiraud^1,2,*^

**Affiliations:**

^1^CAMIN, INRIA, University of Montpellier, Montpellier, France

^2^NEURINNOV, Montpellier, France

^3^University of Montpellier, Montpellier, France

^4^APHP, Paris / CHU Rennes, France

^5^ORTHOSUD, St Jean de Védas, France

^6^Center Bouffard-Vercelli USSAP, Perpignan, France

*To whom correspondence should be addressed.

**Supplementary Materials**

***Subject profiles***

Inclusion criteria were complete motor tetraplegia, age between 18 and 65 years old, neurological stability for at least 6 months, programmed surgery to restore elbow extension, and positive electrical mapping for at least one ﬂexor or one extensor.

| **Subject** | **Age at the inclusion (years)** | **Lesion time (months)** | **Stimulated Arm** | **ICSHT**^^[[1]](#footnote-1)^^ **score** |
| --- | --- | --- | --- | --- |
| **P1** | 37 | 26 | left | 0 |
| **P2** | 37 | 40 | right | 0 |

*Supplementary Table 1. Subject characteristics.*

***Electrode configurations***


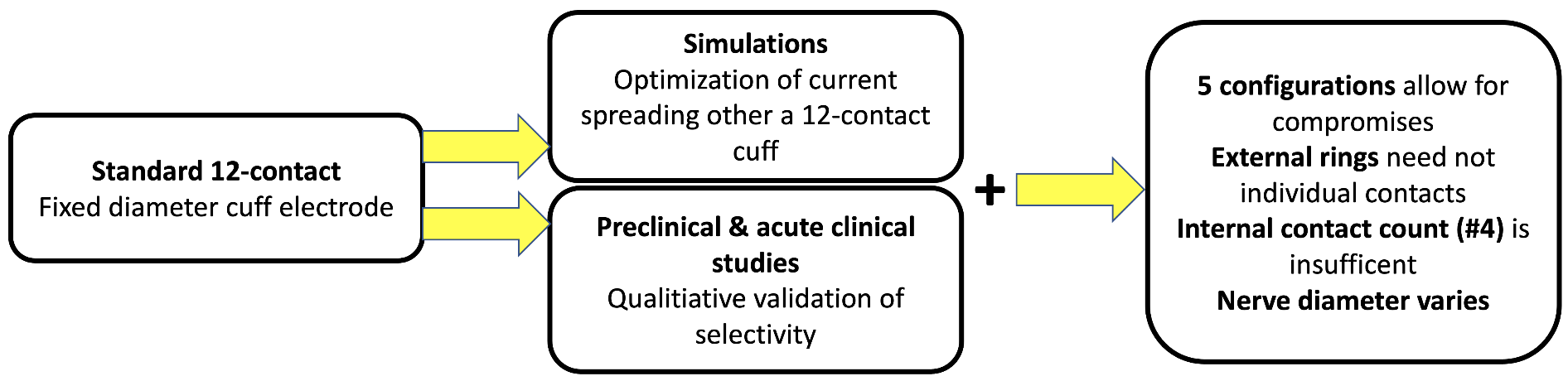


*Supplementary Figure 1: preliminary in-silico and clinical studies using standard state-of the-art multi-contact cuff electrodes and stimulation paradigms.*

As described on the figure Supp. 1, a standard 12-contact cuff electrode was initially used to investigate new paradigms of selective stimulation. All configurations were issued from a theoretical study^36^ in which the optimal current spreading was computed to obtain the best selectivity, efficiency and robustness. Efficiency was defined as the lowest current needed to obtain the highest selectivity, and robustness was defined as the range of current that maintained selectivity with a maximum variation of 50% of the intensity. The wider this range, the more robust the configuration was. As the size of fascicles and the associated functional movements were not known, a large set of selective configurations was tested in our preclinical trial^34^ and acute clinical trial^35^. The simulations provided a subset of 5 optimal configurations. Intraoperative testing provided experimental results among 4 were the same as the optimal theoretical ones. Based on these results, the study presented in this paper was prepared and conducted as shown on the *Supp. Fig. 2*.


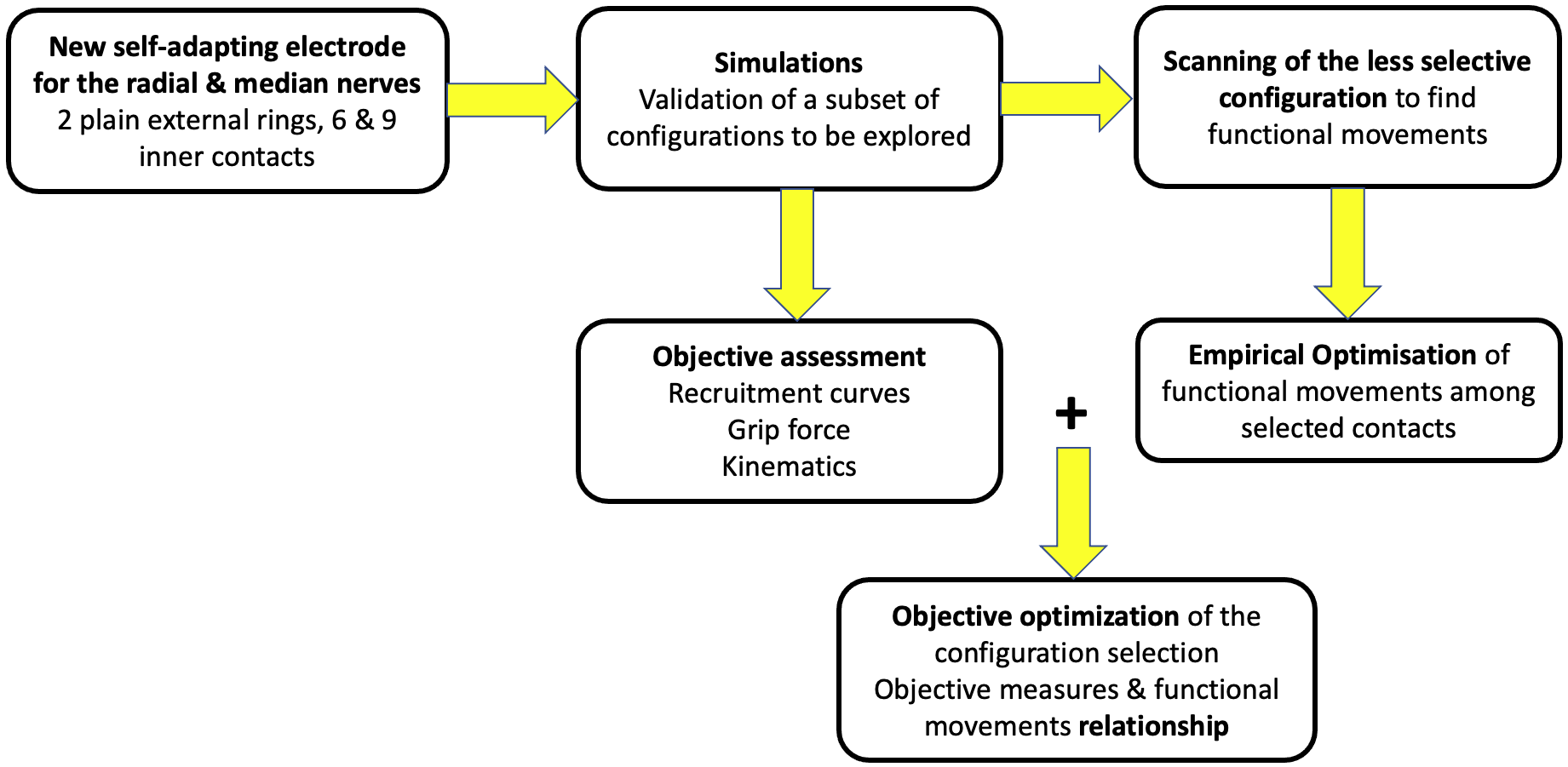


*Supplementary Figure 2: a new design of a self-adapting cuff electrode was produced for the present study. Configurable current sources allow to provide the needed 3D current spreading previously defined adapted to the actual number of usable inner contacts.*

1. A new electrode was designed with the following features: self-adapting size to ensure a good fitting to nerve diameter, external rings without a plurality of contacts as previous studies demonstrated that it was useless, and an increased number of inner contacts from 4 to 6 (radial nerve) and 9 (median nerve).

We limited the search to the following relevant configurations into four groups: TTR for high selectivity with low efficiency, STR for medium selectivity and high efficiency, TLR for low selectivity and high efficiency and Ring for no selectivity as a benchmark. The term selectivity refers to the targeted nerve’s area even though Ring is able to elicit isolated movements. Simulations with the new design (exact sizes of the cuff and its contacts) were performed to validate that design modifications do not change selectivity properties. The nerve was modeled with the parameters used in Dali et al.^36^. The right column of *Supp. Tab. 2* shows activation maps for 0.1 µA and 0.2 µA with the self-adapting electrode considering 8 usable contacts. It shows that the activated areas and their evolution while doubling the intensity are consistent with previous simulations^36^.The 6-contact has a similar activation map. They clearly show increased selectivity with a wider range of intensities from Ring to TTR. It allows to guide the use of configurations from TLR to more accurate but less synergic TTR configuration.

| Name | Distal | **C** | Aadj | Aopp | Aadj | Proximal | Schematic | Simulated maximum activation functions maps (0.1 & 0.2 µA) |
| --- | --- | --- | --- | --- | --- | --- | --- | --- |
| Ring | -14 | **0** | **0** | **0** | **0** | 14 | 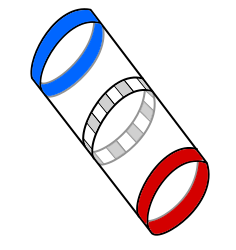 | 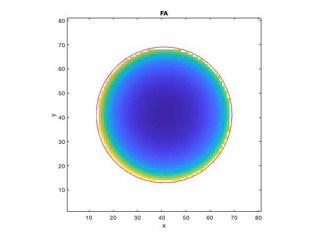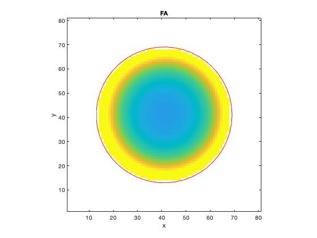 |
| Tripolar Longitudinal + external Rings (TLR) | 7 | **-14** | 0 | 0 | 0 | 7 | 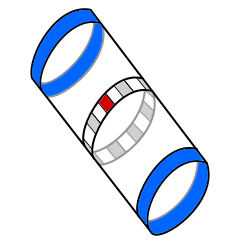 | 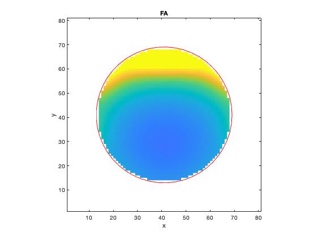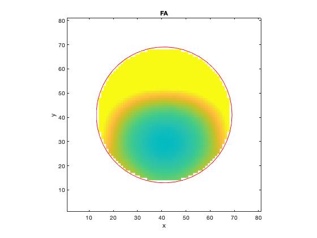 |
| Steering + external Rings (STR) | 3 | **-12** | 0 | 6 | 0 | 3 | 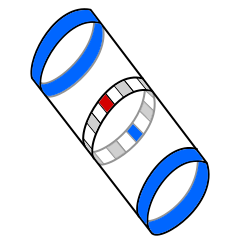 | 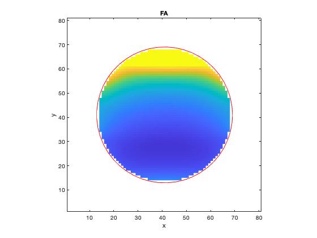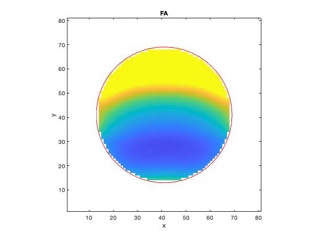 |
| Transverse Tripolar + external Rings (TTR) | 3 | **-12** | 3 | 0 | 3 | 3 | 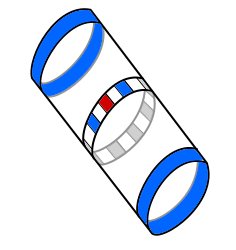 | 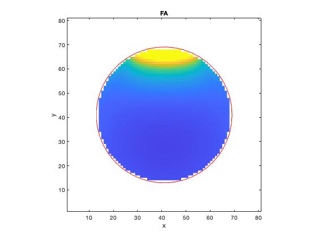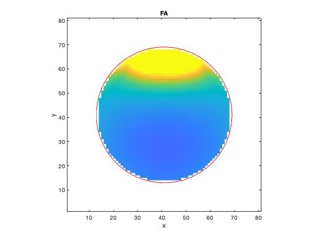 |

*Supplementary Table 2. Stimulation conﬁgurations. One cathode was activated with a ratio of -12/15 (or -14/15). Its position was labeled Ci (1 to 6 for the radial, 1 to 8 for the median). Aopp means the contact in the opposite position to the cathode and Aadj are the 2 contacts apart from the cathode. Ratios are in 1/15. For the electrode drawing, the anode is in blue (hyperpolarization) and the cathode is in red (depolarization).*

During the protocol the electrode’s configurations were scanned following the steps (frequency and pulse width remain fixed):

1. **Systematic scan of TLR configuration (6 resp. 8 cathodic contacts) session**: for each contact the medical doctor assesses the functional outcome while adjusting the intensity. A subset of triplets are selected for which the functional outcome provide one of the 3 targeted movements: intensity amplitude - obtained functional movement – cathodic contact.
2. **Empirical optimization session**: from the previous subset of triplets, STR and TTR configurations are explored to further enhance the quality of the obtained functional movements.
3. **Objective assessment session**: recruitment curves are recorded (all contacts, all configurations) versus intensity. Kinematics and grip forces are measured for the 3 movements with their optimal settings.

***Stability of the settings***

The impedances were quite stable (Supp. Fig. 1, a); the average (standard deviation) of the inner contact impedances varied from 3.92 k𝛺 (±1 k𝛺) at D1 to 4.02 k𝛺 (±0.79 k𝛺) at D27 while the average impedance for the 4 distal rings varied from 1.57 k𝛺 (±0.524 k𝛺) to 1.17 k𝛺 (±0.231 k𝛺). Standard deviations decreased over time so the impedances tended to stabilize around a restricted bounded interval. Among the 4 implanted cuff electrodes that represent 8 rings and 30 central contacts only 2 were broken. They were immediately detected as open circuits and were not used later (P1, Radial nerve contact 1; P2, Median nerve contact 2). They were probably damaged during the implantation surgery. No other contact showed failures.


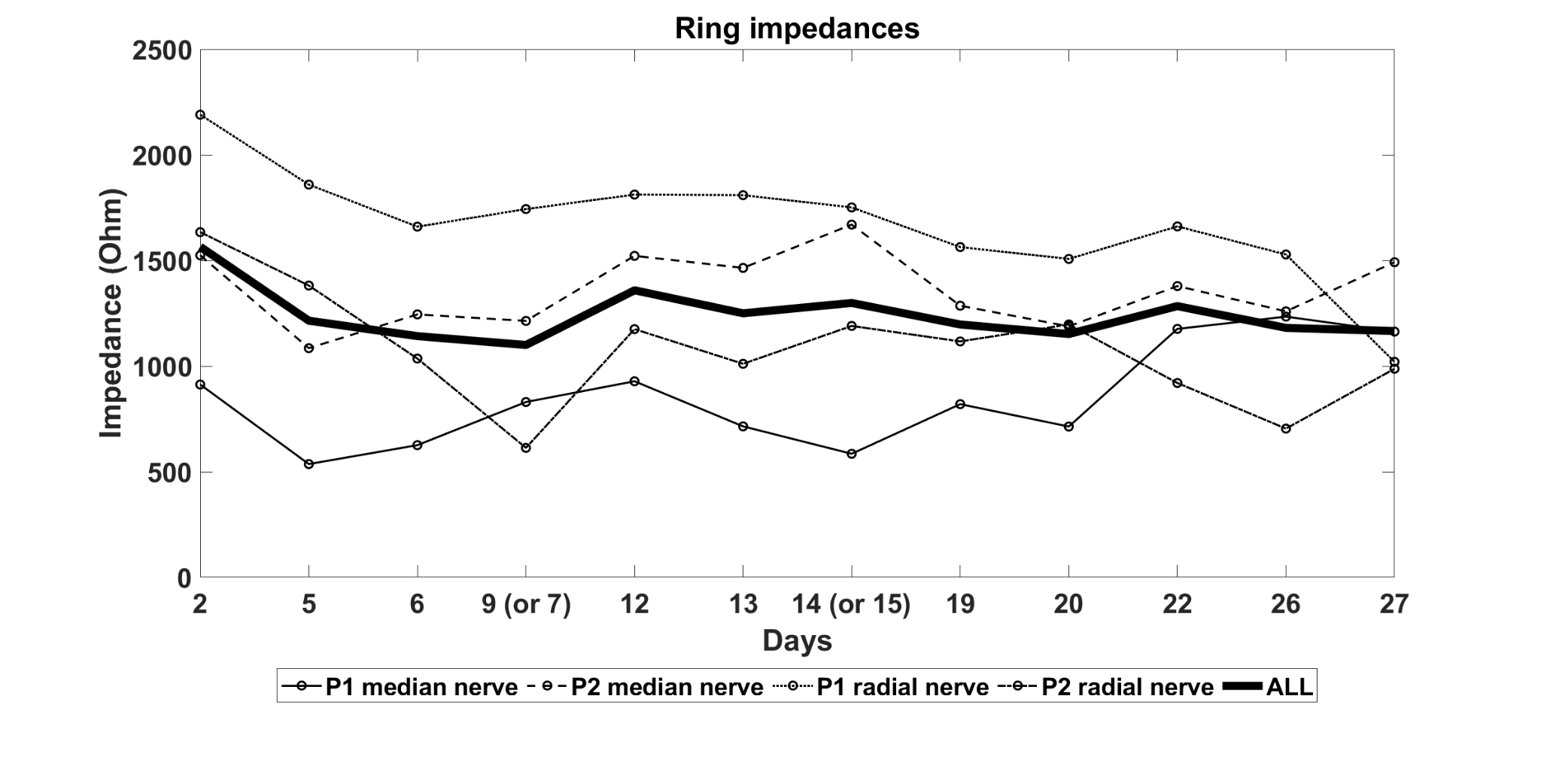

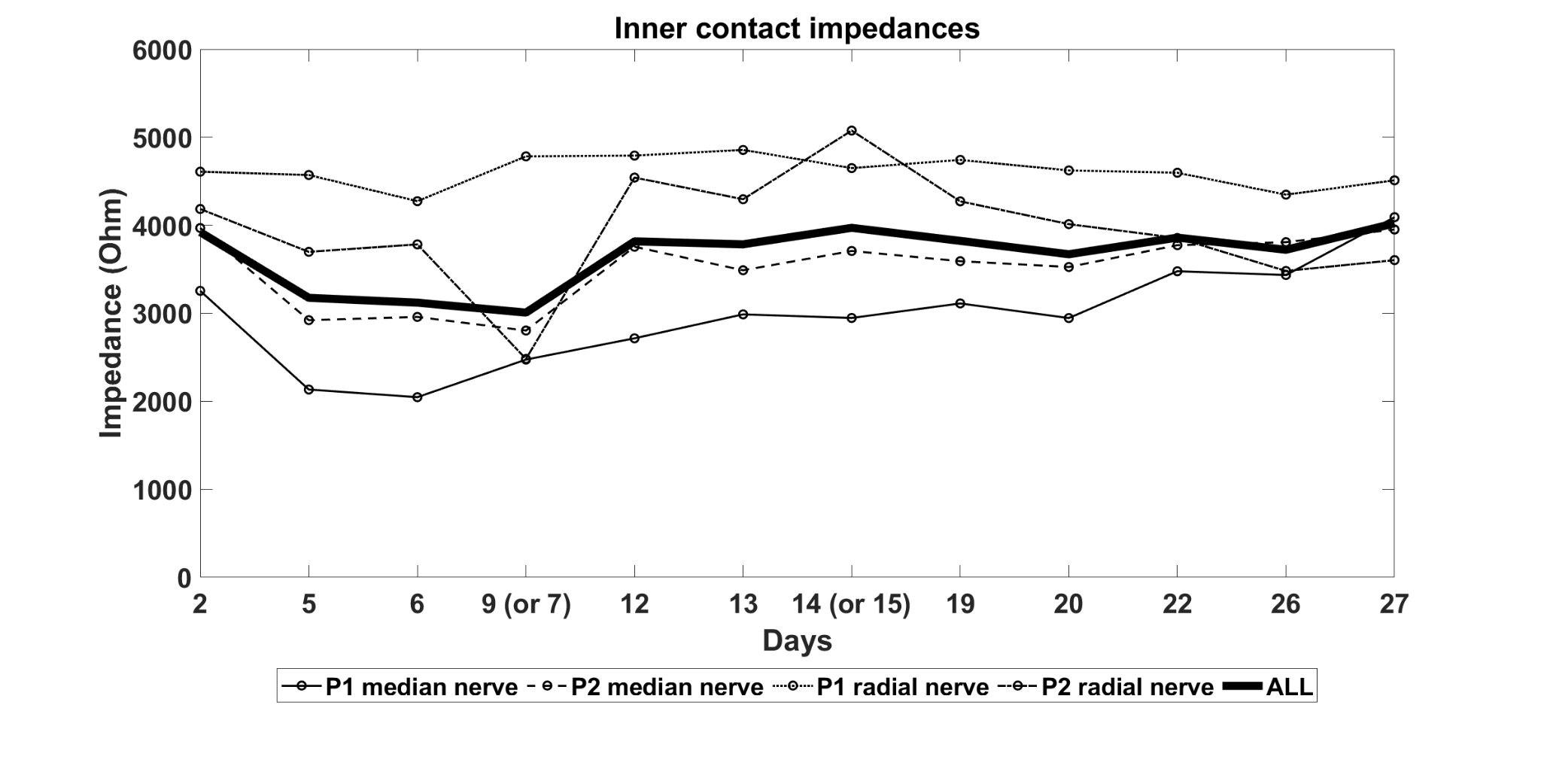


*Supplementary Figure 3: impedances of contacts and rings over time.*

The stimulation thresholds of all the contacts of the 4 electrodes followed along the 28 days remained stable with no increase or decrease during the whole 28 day period in which the protocol took place. Indeed, the settings were very reliable from day to day so that we never had to change these optimal settings.


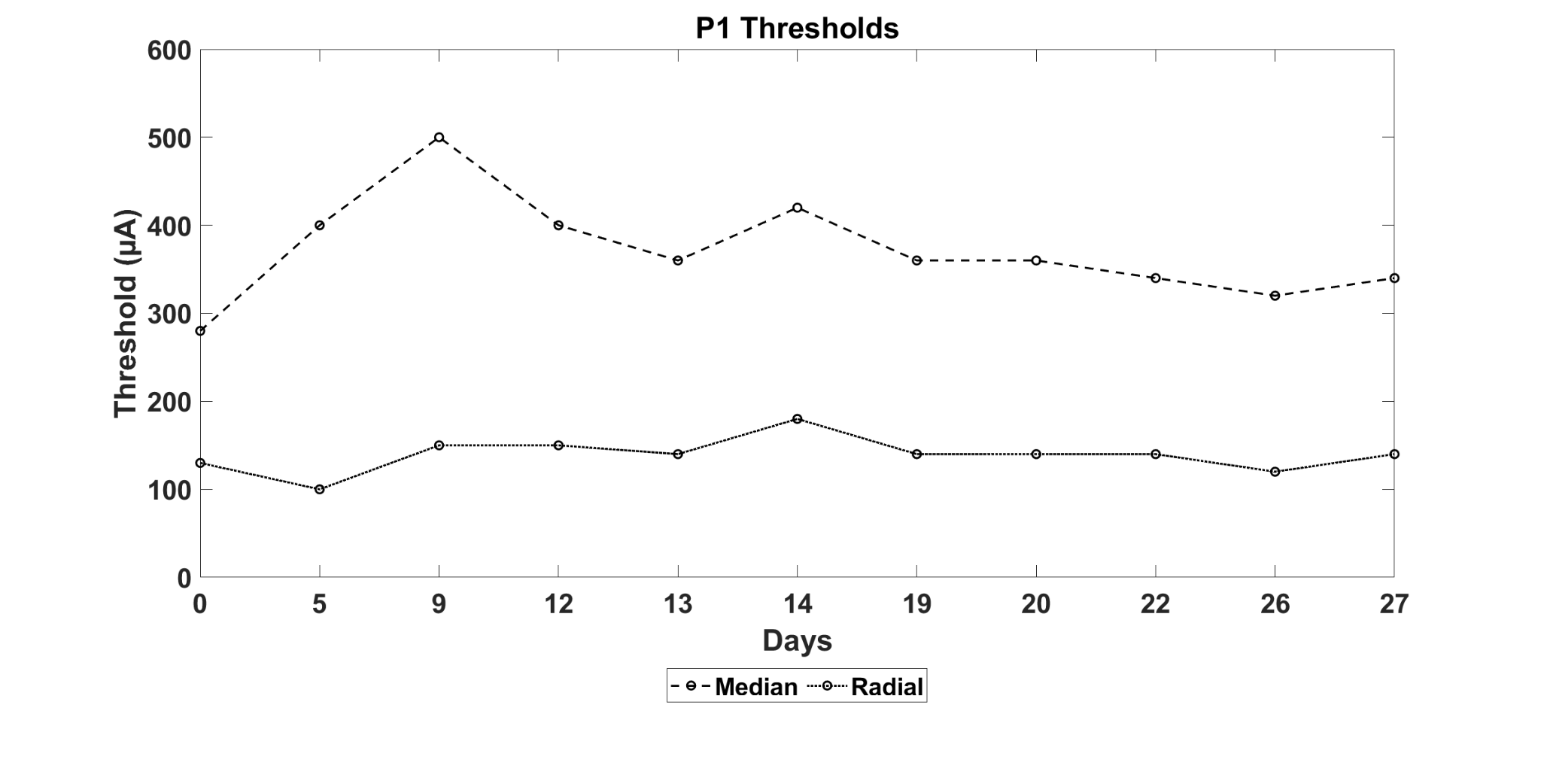

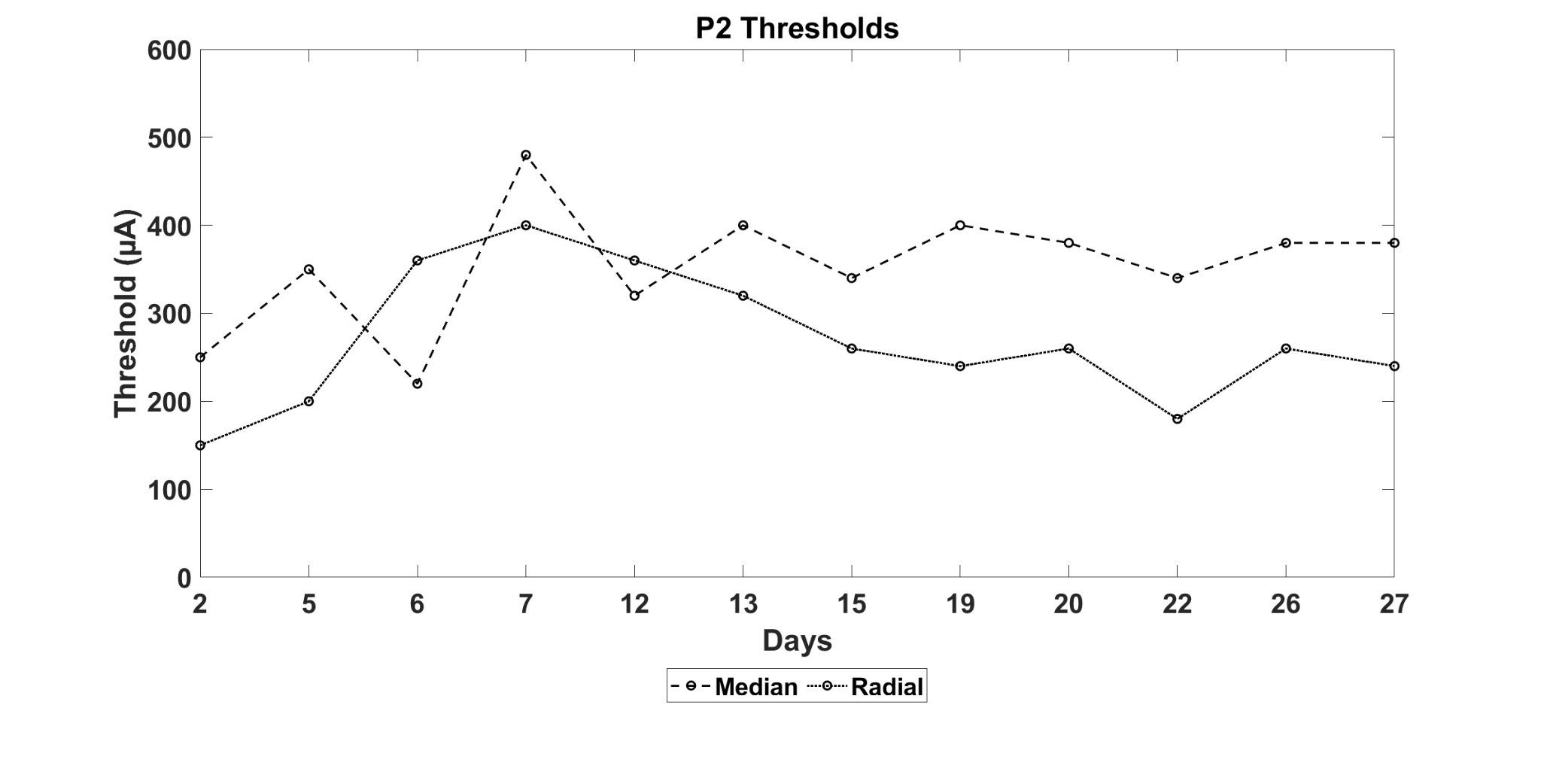


*Supplementary Figure 4: a) stimulation threshold using the standard bipolar configuration. b) mean intensity threshold in µA for a recruitment greater than 0.1 by muscles for each type of configurations (TLR, STR and TTR)*


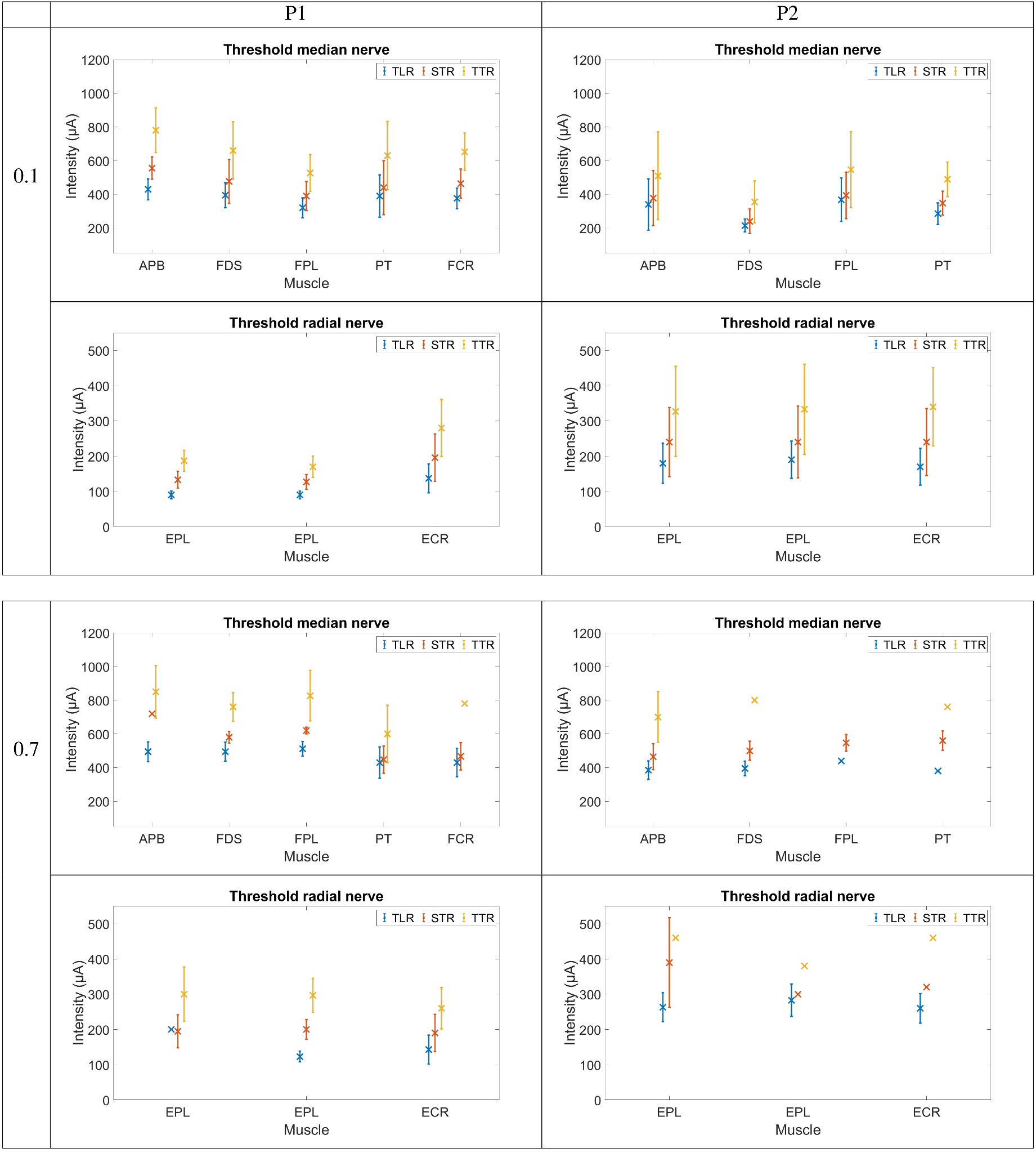


*Supp. Fig. 4 (a, b)* reports the thresholds obtained for each muscle, for each configuration and for each contact. It shows that the values are increasing while using more selective configurations as the theory predicted. The mean threshold for the median nerve is higher than the mean threshold for the radial nerve for both participants. It could be explained by the fact that the radial nerve has a smaller diameter and thus its depth is lower, so the need for high intensities.


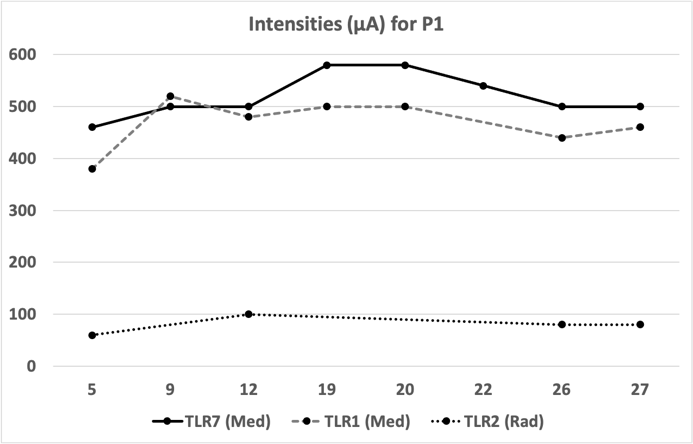

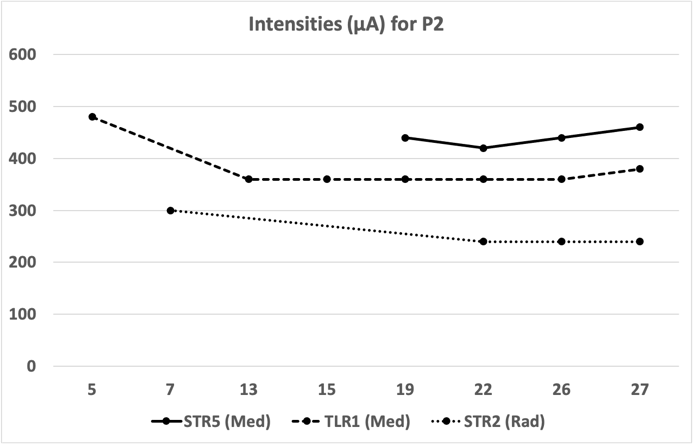


*Supplementary Figure 5: The stimulation intensity settings along the clinical trial from D5 to D27 post surgery with the same configurations for each movement.*

*Supp. Fig. 5* shows the intensities settings for the 3 configurations, for each patient, that have been finally selected at the end of the protocol as the best ones (best obtained functional movements). The settings are stable over time as the amplitude variation during the last week (from about D20 up to D27) is 0 µA for radial nerve on both patients, maximum 40 µA maximum (2 steps <10%) for the median nerve for both patients.

***Recruitment curve’s details***


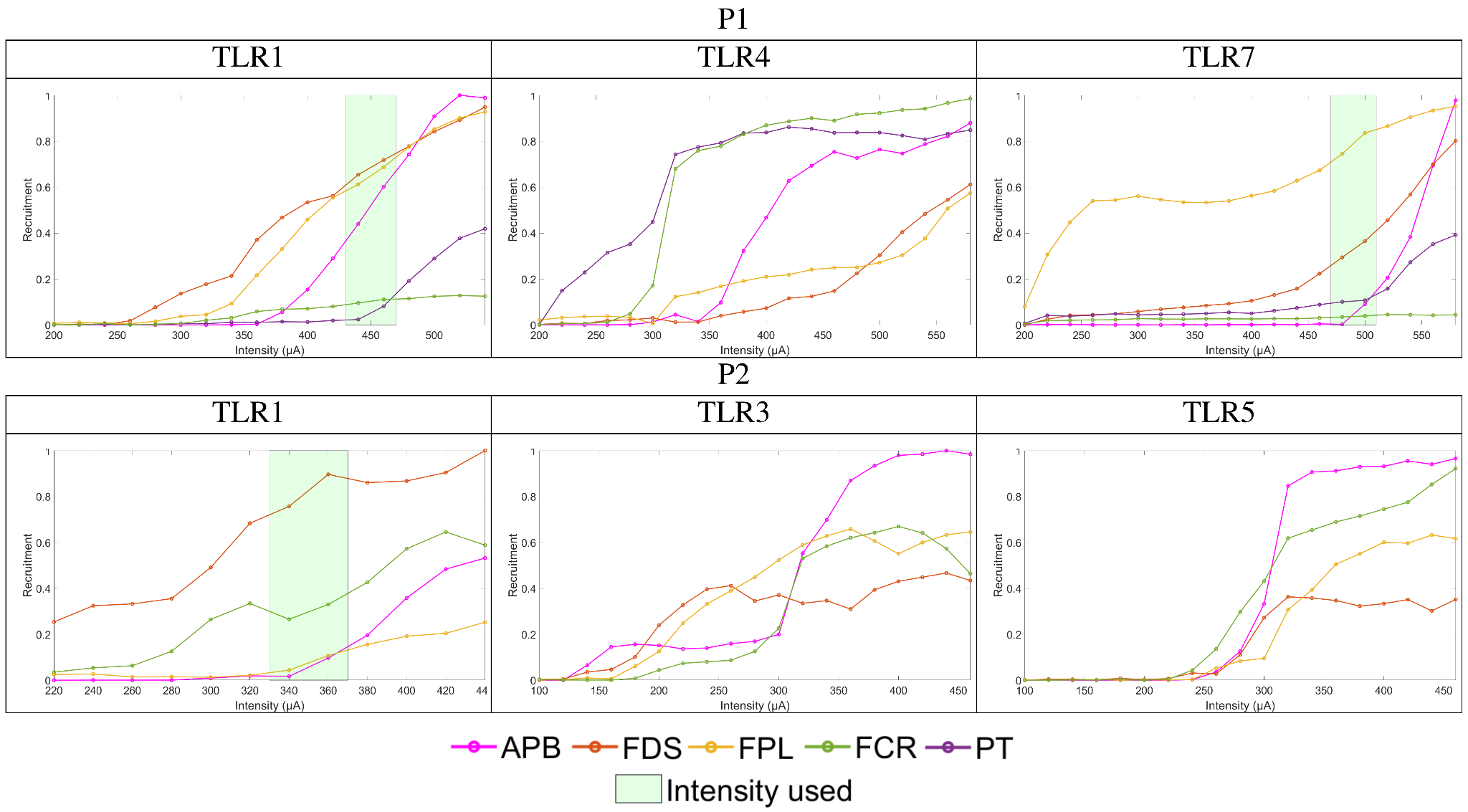


*Supplementary Figure 6: 3 examples of recruitment curves for different contacts, configuration TLR, on the median nerve of both participants. The sequence of activation of muscles differs depending on the selected contact as the cathode.*


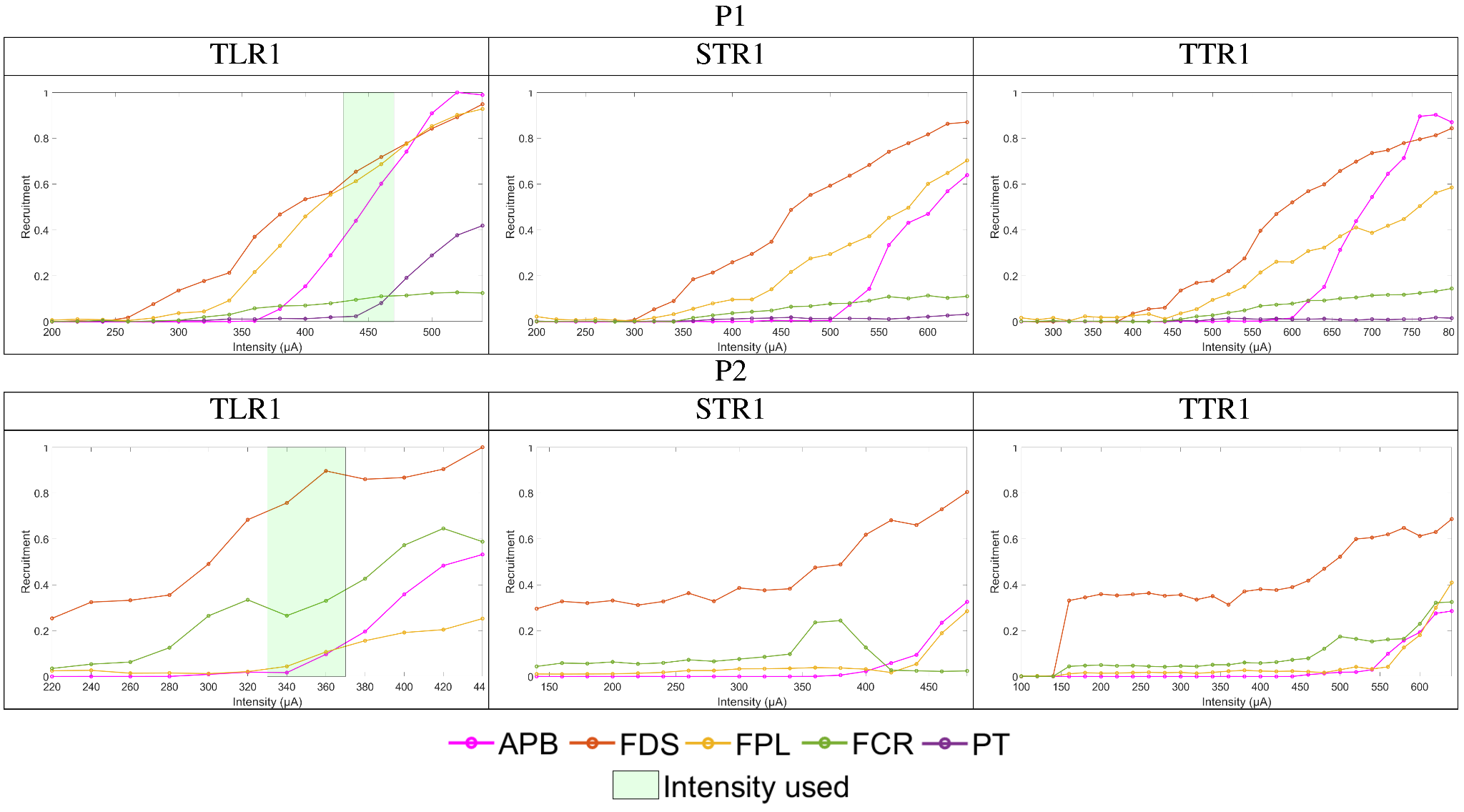


*Supplementary Figure 7: examples of recruitment curves for the same contact, different configurations, on the median nerve of both participants*


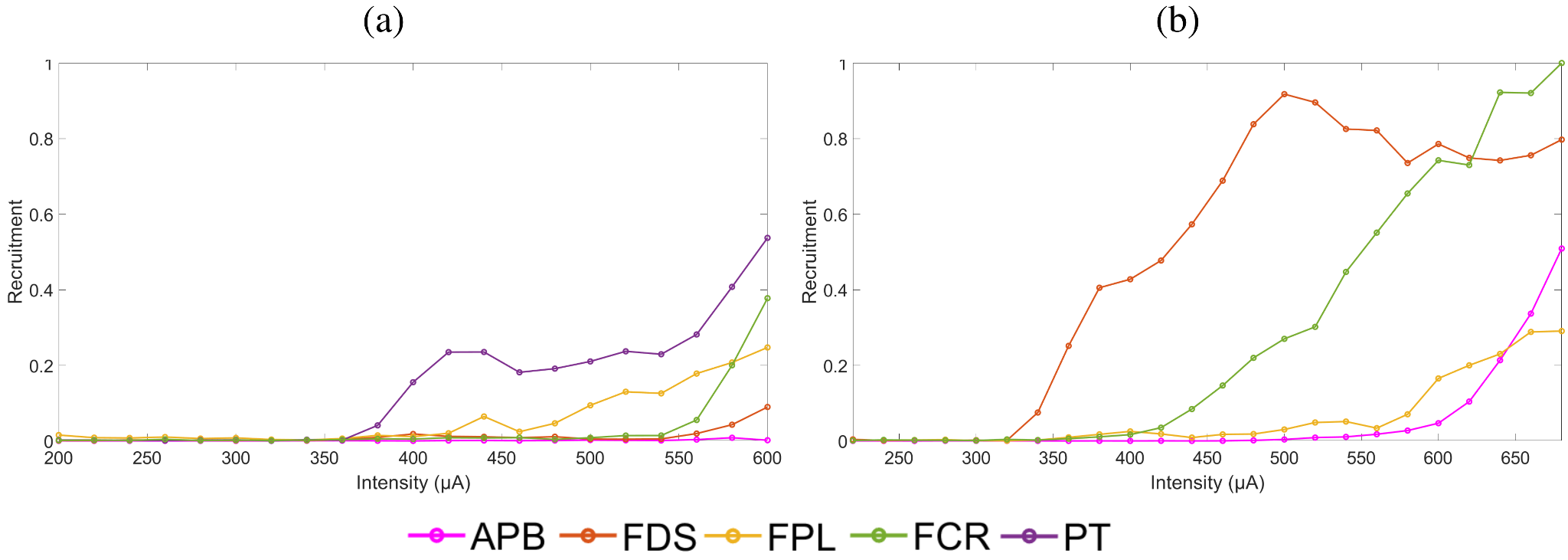


*Supplementary Figure 8: Recruitment curves for the ring configuration, (a) P1 and (b) P2.*

*Supp. Fig. 6* demonstrates that changing the contact while keeping the same current distribution (TLR) induces different muscles’ recruitment orders. This order is not always maintained when the intensity is increased. This is due to the unknown fascicle organization, but different grasping are generated depending on the contact. *Supp. Fig. 7* shows that the recruitment is similar while the configuration is changed, supporting the fascicular organization, but with an extended range of intensities between the successive recruitment of muscles. The TTR allows for a higher accuracy of muscle’s activation balance but to the price of a global increase of the intensity. TTR allows for far fascicles (from cathode) to be avoided thus limiting the activation of the corresponding group of muscles. *Supp. Fig. 8* shows that a classical bipolar configuration provides a given recruitment order that is very different between both patient and cannot provide a useful grasp.


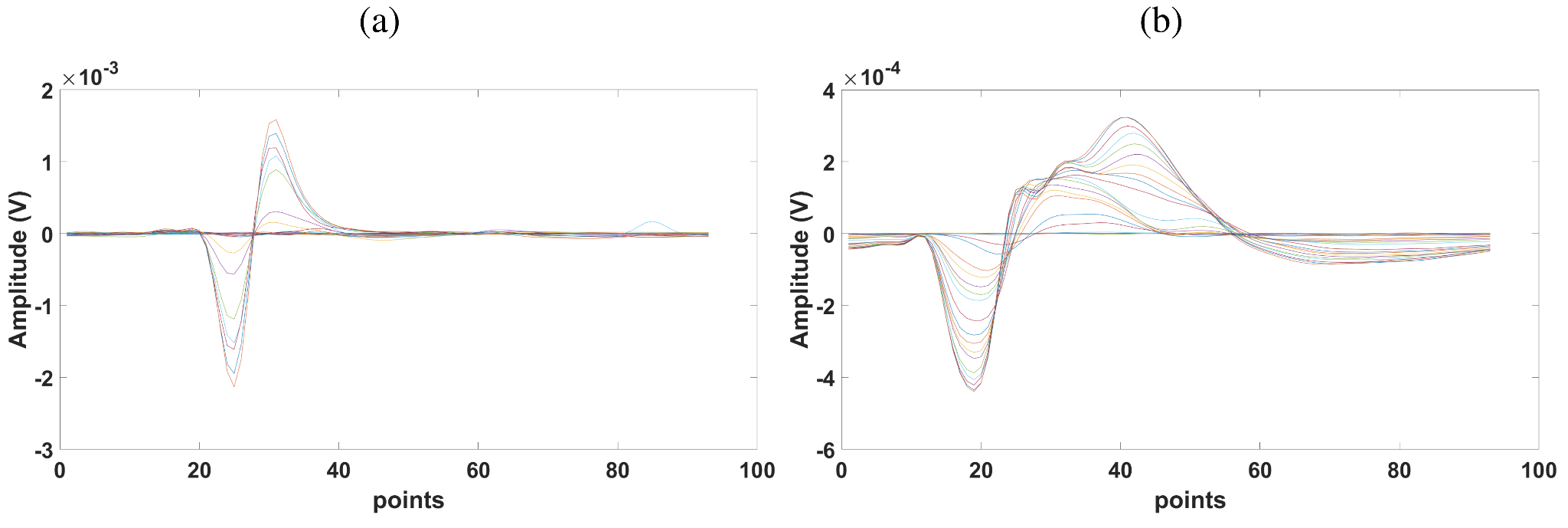


*Supplementary Figure 9: M-Waves (single* ***APB*** *response (a) and combined response (****FDS*** *and* ***FCR****) (b), both recorded with configuration STR1 with increasing intensities.*

*Supp. Fig. 9* shows the problem of composed M-waves (b). Indeed the temporal course cannot be processed so both components can be clearly separated or even quantified in a robust way. No pure M-wave are available so template matching is not useable and, as both M-waves evolve while the current intensity increases, they are not fully independent so ICA and PCA fail. However, both M-waves has a different time-frequency span so the Meyer wavelet transform followed by a selection of a non-overlapping time-frequency area representing each M-wave successfully allows for computing associated recruitment curves^39^. The method is detailed in the *Supp. Fig. 10.*


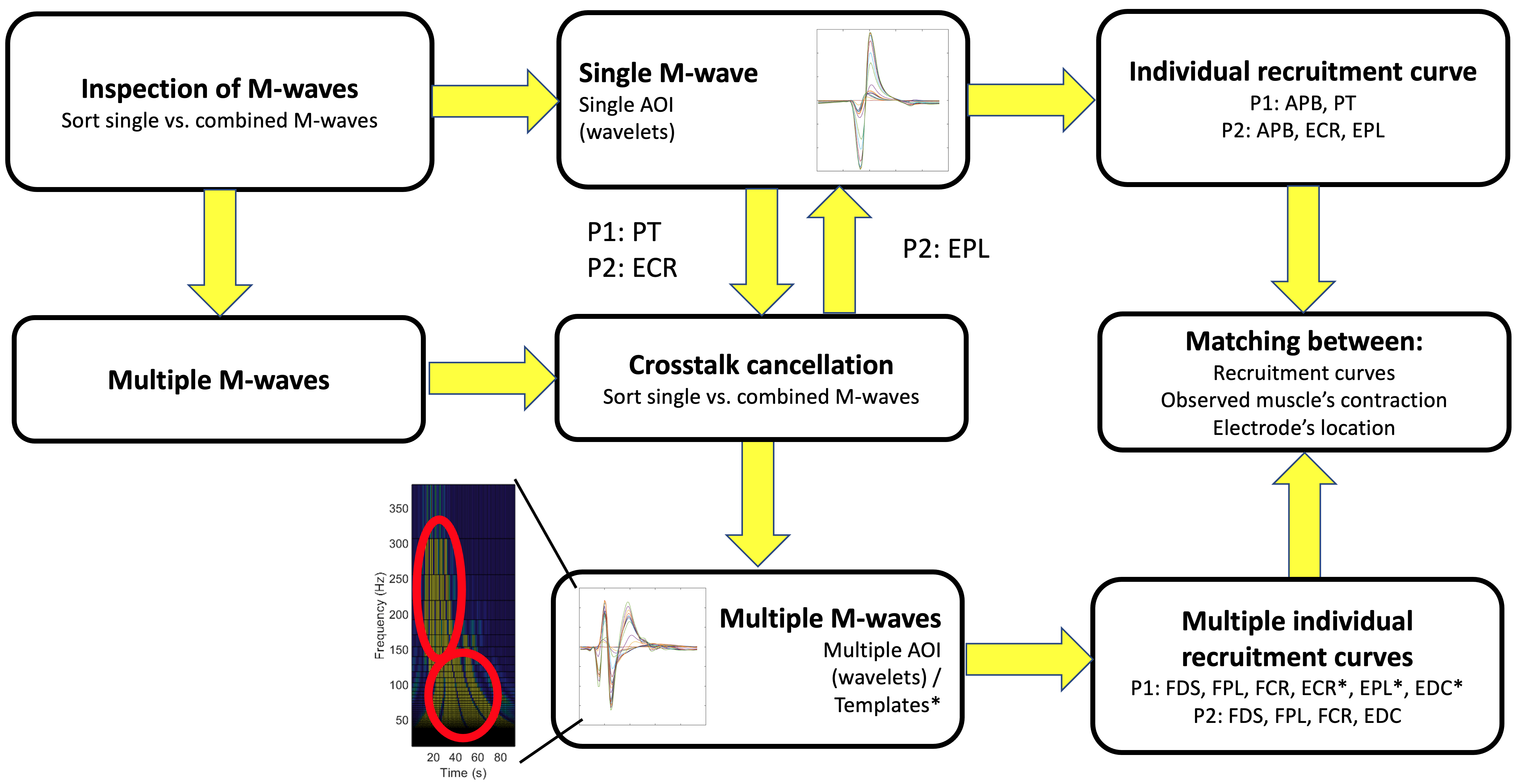


*Supplementary Figure 10: method of M-wave sorting and crosstalk cancelation.*

Data is recorded on 8 channels for muscles innervated by the median nerve (**APB, PT, FDS, FPL, FCR**+ 3 redundant channels) and 4 channels for muscles innervated by the radial nerve (**ECR, EPL, EDC**+ 1 redundant channel). The method follows the steps:

1. Sort channels with a single M-wave => extract recruitment curve based on a single Area of Interest in the time frequency domain.
2. Substract single M-wave after time and amplitude adjustments on other channels (cross talk cancellation).
3. New sorting of channels with a single M-wave => extract recruitment curve based on a single Area of Interest (AOI) in the time frequency domain.
4. Remaining 2 combined M-waves channels => extract recruitment curves with specific AOI.
5. Check consistency between recruitment curves, electrode’s location used for extraction and observed movement (muscle’s contraction).

For P1 radial nerve stimulation (* mark on *Supp. Fig. 10*), the multiple M-waves were very difficult to extract following this method so a template matching with 3 synthetic M-waves (gaussian like) was applied successfully. The method was not fully validated but consistency between recruitment’s curves, EMG electrodes’ location and observed induced movement was positively checked.

***Advanced posture management with co-contraction***

To obtain an efficient grasping, the wrist joint control is mandatory; as mentioned in the introduction, with FES external stimulation, it is a major limitation so that either an orthosis or a voluntary control of the wrist is needed. In our current study, we faced the same problem and we used an orthosis. Without wrist control, either with an orthosis or an active wrist extension, the grasping occurs but with much less efficiency (unrecorded data). We tested on patient P1 an active wrist extension without orthosis through the radial stimulation. We selected a configuration for which thumb and fingers extensions were minimal, to avoid counteracting the stimulation of flexors, and **ECR** activation was maximal (*Supp. Fig.* 11).


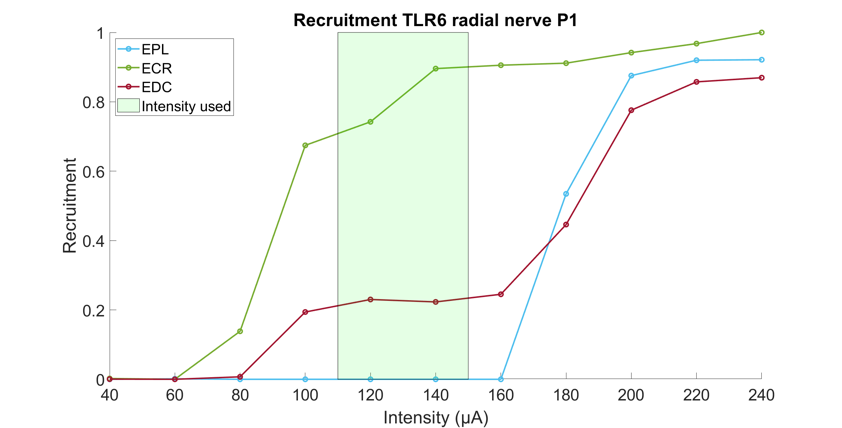


*Supplementary Figure 11: TLR6 at 140 µA shows a strong contraction of the wrist extensor (****ECR****) without* ***EPL*** *and with a weak* ***EDC*** *activation (0.2) (Frequency 24 Hz, Pulse Width 150 µs).*

Compared to Figure 2, where the stimulation of the radial with TLR2 configuration activates all muscles (between 80 and 100 µA), TLR6 allows to isolate **ECR**. This a key advantage of our selective approach for which we can choose between synergies or an isolated muscle’s activation. To further increase the efficiency of the grasping, the **ECR** was activated 500 ms prior to the grasping movement keeping the **ECR** activated. Indeed a simultaneous activation was less efficient demonstrating that the initial posture of the wrist is very important and should be extended before the grasping is engaged. The comparison of the kinematics between both strategies are illustrated on *Supp. Fig. 12*. The obtained final movements are different even though the flexors’ activations are identical. The functional grasps were however similar but it shows that the kinematics depends on not only the activations, so the stimulation parameters, but also the global biomechanical state, in particular the initial posture of the wrist. These findings are in accordance with Dunn et al.^8^ who reports that wrist extension participates to the efficiency of the grasp.


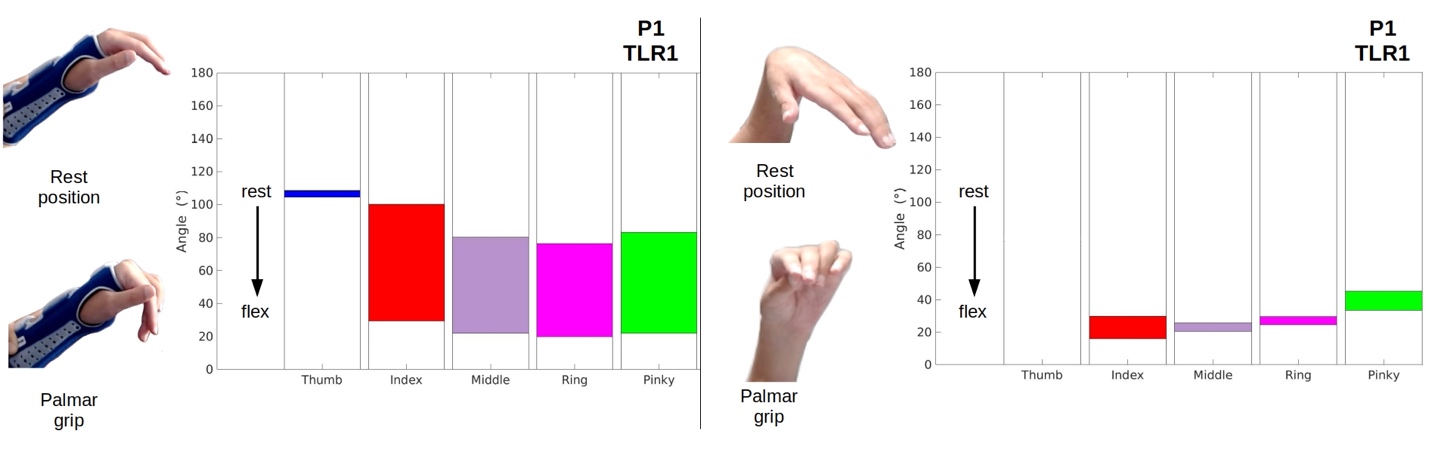

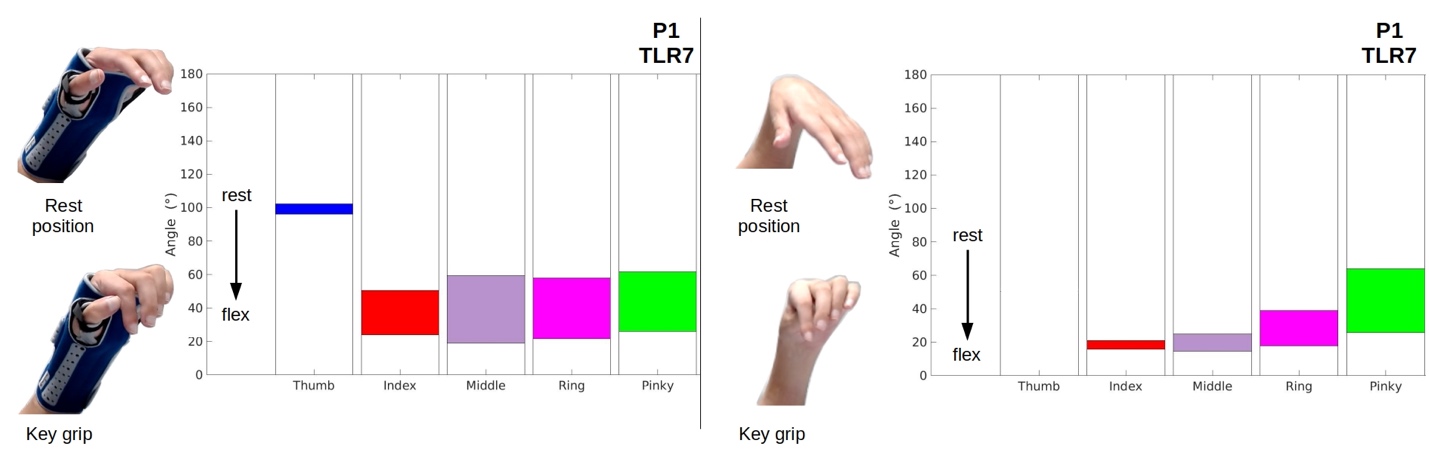


*Supplementary Figure 12: P1 grasps with the orthosis (left) and co-contraction (right) of* ***ECR*** *(TLR2 used for the radial nerve stimulation).*

Kinematics shows that without wearing a wrist orthosis and with **ECR** activation instead, the final flexion of all fingers and the thumb are greater, except for the ring and pinky. Indeed, **FDS** is not supposed to flex Ring and Pinky explaining that there is no difference as they are probably flexed due to some mechanical coupling. The rest position is obviously very different with and without wrist orthosis. We can notice that the orthosis does not guarantee a constant rest position for fingers as they are free to move. Thumb is partly constrained so its flexion is limited compared to free moving thumb. Moreover, the thumb final position while the wrist is actively extended is more opened than at rest (not represented on *Supp. Fig. 12*). It means that the biomechanical balance is not the fully flexed thumb however providing a strong thumb rigidity due to concomitant extension of the wrist.

| P1 | With Orthosis | With Co-contraction | ∆ Kinematics |
| --- | --- | --- | --- |
| Rest: Key Grip  Rest: Palmar Grip  *∆ Rest* | 102° 51° 59° 58° 62°  109° 100° 80° 76° 83°  *7° 49° 21° 18° 21°* | 45° 21° 25° 39° 64°  46° 29° 26° 30° 45°  *1° 8° 1° -9° -19°* | -57° -30° -34° -19° 2°  -63° -71° -54° -46° -38° |
| Key Grip  Palmar Grip | 96° 24° 19° 22° 26°  105° 29° 22° 20° 22° | 50° 16° 14° 18° 26°  75° 15° 21° 25° 33° | -46° -8° -5° **4° 0°**  -30° -14° -1° **5° 11°** |
| ∆ Key Grip  ∆ Palmar Grip | -6° -27° -40° -36° -36°  -4° -71° -58° -56° -61° | **5°** -5° -11° -21° -38°  **29°** -14° -5° -5° -12° |  |

*Supplementary Table 3: comparison of angles (Thumb-Index-Middle-Ring-Pinky) obtained with orthosis or with co-contraction (****ECR****).*

1. The ICSHT classiﬁcation is used to list active muscles below the elbow (allowing an active movement against gravity and resistance) in patients with tetraplegia. [↑](#footnote-ref-1)
