# Supplementary material for: Activating effective functional hand movements in individuals with complete tetraplegia through neural stimulation
Source: Sci Rep. 2022 Oct 6;12:16189. doi: 10.1038/s41598-022-19906-x (PMC9537317; doi:10.1038/s41598-022-19906-x)
Supplement: Supplementary file 3 — Supplementary Information 2. [file 41598_2022_19906_MOESM3_ESM.docx]

**Activating Effective Functional Hand Movements in Individuals with Complete Tetraplegia Through Neural Stimulation**

**Authors:** Christine Azevedo Coste^1,*^, Lucie William^1^, Lucas Fonseca^1^, Arthur Hiairrassary^1,2^, David Andreu^2,3^, Antoine Geffrier^4^, Jacques Teissier^5^, Charles Fattal^1,6^, David Guiraud^1,2,*^

**Affiliations:**

^1^CAMIN, INRIA, University of Montpellier, Montpellier, France

^2^NEURINNOV, Montpellier, France

^3^University of Montpellier, Montpellier, France

^4^APHP, Paris / CHU Rennes, France

^5^ORTHOSUD, St Jean de Védas, France

^6^USSAP, Perpignan, France

*To whom correspondence should be addressed.

AGILIS Clinical Trial was following ISO 14155 « Good Clinical Practice » recommendation. The paper reports part of the results of the clinical trial. This synopsis sums-up the essential points of the protocol.

| Title | Functional evaluation of the restoration of prehension in patients with tetraplegia by implanted neural stimulation |
| --- | --- |
| Acronym | **AGILIS** |
| Registration | Database: ClinicalTrials.gov  Registration number: NCT04306328  First registered date: 12/03/2020 |
| Sponsor | **AP-HP,** Paris, France |
| Investigation centres | USSAP rehabilitation centre, Perpignan, France: responsible for the patient follow-up during the 28 days  PI: Dr Charles Fattal.  Saint Jean clinic, ORTHOSUD, Saint Jean de Védas, France: responsible for the implant and explant surgeries of the electrodes  PI: Dr Jacques Teissier. |
| Other partners | INRIA, Montpellier, France, Mrs Christine Azevedo Coste  NEURINNOV, Montpellier, France, Mr David Guiraud |
| Principal Investigator | **Dr Charles Fattal**, MD-PhD, USSAP, Perpignan, France, responsible for patient’s recruitment. |
| General scientific rationale | Electrical muscle stimulation has been used for decades in rehabilitation units dedicated to persons with spinal cord injuries. It has been shown to be effective in preventing muscle atrophy following spinal cord injury. It can also be used to decrease spasticity and especially to promote functionally useful motor control. This is the so-called Functional Electrical Stimulation (FES). In the person with tetraplegia marked by a severe motor deficiency of the upper limbs, FES is today the only technique allowing to consider a functional restoration of the gripping movements in the case where the active muscular resources below the elbows are absent or insufficient to allow tendon transfer surgery (groups 0, 1 and 2 of the ICSHT classification). The application of FES to the upper limb of the person with tetraplegia was established in the 2000s with the first implanted electrical stimulation neuroprosthesis called "Freehand" with 8 to 12 electrodes implanted in individual targeted muscles. The discontinuation of its marketing in 2001 deprived many patients of a very promising functional solution, which has not yet been replaced.  Like the "Freehand", all the devices using FES directly stimulate the muscles (surface, intramuscular or epimysial electrodes) and therefore require a high number of internal components with a theoretical risk of infection and greater risk of rejection, since each muscle must be activated *via* an electrode. Selective neural stimulation as we propose, allows to stimulate several muscles *via* a single electrode. Moreover, neural stimulation requires less energy for muscle activation. In our approach, 2 electrodes will be implanted above the elbow on the median and radial nerves. This considerably reduces the number of elements implanted and therefore i) the risk of infection, ii) the risk of failure, iii) the surgical risk thanks to a limited surgery. The promises of such an approach have been validated in a first feasibility study on 9 patients with tetraplegia (Tigra et al., JNER, 2020, cited in the main file). The procedure consisted of placing a multi-contact cuff electrode either around the radial or the median nerve and observing the effects of neural electrical stimulation in terms of muscle selectivity, force produced and movement induced. This procedure has been applied intraoperatively in subjects with tetraplegia undergoing active elbow extension restoration surgery, with an open approach allowing direct access to the radial nerve or the median nerve depending on the subject. The electrodes were tested intraoperatively and removed immediately after being tested. In other words, they were left in place for 30 to 45 minutes. The summary of the results of this first study are:  - No adverse effects were noted.  - No failure of the electrodes was detected.  - For all the subjects, it was possible to selectively stimulate muscle groups to obtain opening of the thumb and fingers, or flexion of the thumb, fingers and obtaining possibly functional gripping such as the gripper with opposition of the thumb or palmar grip.  On the basis of the results, we take a further step in the development of a grip support device for patients with spinal cord injuries:  - by proposing the implantation of two cuff electrodes in the arm. The electrodes will be kept in place for a period of 1 month before being explanted. A percutaneous cable will connect the electrodes to an external connector.  - by offering a piloting interface allowing subjects to trigger programmed stimulation sequences applied to the implanted electrodes from voluntary movements or muscle activities of segments above the lesion to perform an opening or closing of the hand adapted to the targeted grip. The stimulator will not be implanted and will be connected to the electrodes connector through the percutaneous cable.  - by combining these two elements with an analytical and functional evaluation of the movements of the hand produced, over a period of 1 month post-implantation in a functional rehabilitation centre.  Our main hypothesis is that multipolar neural electrical stimulation of the median nerve (flexion) and the radial nerve (extension) allows:  - on the one hand, selective, individualized motor activation (muscle by muscle)  - on the other hand, a synergistic motor activation (association of several muscles) for the production of functional movements. |
| Specific scientific rationale | The paper deals with part of the clinical trial and thus described more in details the scientific rationale. This rationale together with the references are given in the main file (see introduction and references sections). |
| Primary objective and endpoint | **Primary objective**  Implantation of 2 epineural multi-contact electrodes around median and radial nerves maintained during 28 days and connected on demand to an external electrical stimulator. Evaluation of the stimulation configurations inducing an individualized and selective motor control of the different motor fascicles in the forearm and hand (fingers, thumb).  **Primary endpoint**  The main endpoint is the selectivity index of the configurations (active electrode contacts and stimulation parameters) which allow individualized recruitment of the different muscles. This will result in the combination of two calculated variables:  • the increase in the RMS value -Root Mean Square- of the corresponding EMG compared to its resting value is greater than 10% of the maximum EMG measured for this muscle. For information, the RMS value is a variable calculated from the raw EMG measurement collected. The stimulation configuration is considered to be selective from the point of view of muscle recruitment when the variation in the RMS value of this muscle accounts for at least 70% of the variation in all the muscle contractions observed.  • the 10% variation of the maximum stroke of the segment considered in relation to the rest position. The stimulation configuration is considered to be kinematically selective when the variation in the position of the segment considered (mm) accounts for at least 70% of the cumulative variation in the variation of all the positions observed. |
| Secondary objectives and endpoints | **Functional objectives**   - assess the patient's ability to perform functional grips (grasp, move, release) of separate objects using multi-contact neural stimulation of the median and radial nerves. - study the functional relevance of neural implantation via the production of 4 movements considered useful: on the one hand, an opening of the 5 fingers and on the other hand in association with an extension of the wrist, a finger-palmar grip with thumb, a finger-palmar grip without thumb and an end-to-side thumb index grip - test two methods of autonomous control of the device by the patient to carry out the different object grips   **Technical objectives**   - determine the optimal strategies for a functional synergic control - evaluate the change in the quality of the stimulation over time (stability of the settings and of the tissue / electrode interface)   **Safety and acceptability objectives**   - monitor local (skin and algological) and general tolerance - monitor the acceptability of the implantation procedure and post-implantation training   **Secondary endpoints**  For Functional objectives:   - The analytical approach will be based on the motor testing of the Medical Research Council (MRC) of each of the muscles stimulated with the aim of evaluating the maximum strength obtained on a scale of 0 to 5 (0 = no movement, 1 = contraction without movement, 2 = full movement without gravity, 3 = full movement against gravity, 4 = movement against slight resistance, 5 = movement against strong resistance). MRC motor testing is a very common test in clinical practice in physical medicine. It will be performed by the doctor. This is an extrapolation since MRC testing is in essence an assessment of muscle strength produced on purpose. This testing will be completed by the evaluation of the clamping forces around an instrumented bullet. - The functional approach will consist of evaluation of the control methods: during the study of the methods of autonomous control of the device by the patient to carry out the different object grips, an experimenter will indicate to the participant which hand movement he must trigger (thumb grip - end-to-side index finger, finger-palmar grip without the thumb, finger-palmar grip with thumb, opening of the 5 fingers), the patient will have to contract certain supra-lesional muscles or mobilize certain segments under voluntary control to signal an order to the neuroprosthesis in accordance with the method chosen. Each movement will be presented 10 times at random to the patient. For each movement, the experimenter will note whether the participant has indeed succeeded in triggering the corresponding movement. For each modality and type of movement, a score out of 10 will be defined, corresponding to the number of successful attempts.   For Technical objectives:   - For each movement the optimal configuration will be retained. This involves determining the optimal strategies for a synergic functional control: stimulation configuration corresponding to the desired functional grips (opening of the 5 fingers, end-to-side thumb-index clamp, finger-palmar grip with thumb, finger-palmar grip no inch) for minimum injected current load. - For each movement, the minimum stimulation intensity to produce the movement will be noted. This involves studying the evolution of the stimulation intensity required to trigger a specific movement with a given stimulation configuration. - The state of contact between the poles of the electrodes and the nervous tissue will be assessed by measuring the impedance of the electrode contacts. This assessment is part of the procedure for verifying the absence of technical failures and maintaining the nominal performance of the neuroprosthesis.   For acceptability and safety objectives   - Local tolerance will be monitored daily after implantation   from the cutaneous point of view, to search for a dermatological lesion of the allergic and / or infectious type at the implantation site. The answer provided daily will be binary yes / no  from an algological point of view, to assess the emergence or worsening of pain   - the number of patients who presented at least one pain at the implantation site, qualified as neuropathic or nociceptive and quantified as ≥ 3/10 using a numerical scale from 0 to 10. - the average intensity of daily pain measured by a numerical scale from 0 to 10. - A baseline assessment before implantation will be performed then every day thereafter for 30 days after implantation and then 15 days after explantation.   The general tolerance will be evaluated on D0 + 29 by the number of patients having presented at least one adverse event during surgery or after implantation. The frequency and type of event must also be specified.  The acceptability of the implantation procedure and post-implantation training will be studied through a questionnaire specifically designed for this protocol –cf. annex- and offered once a week. Its assessment will be renewed on D + 45. |
| Experimental scheme | Prospective, proof-of-concept exploratory case-series study carried out in patients with spinal cord injury, tetraplegic neurological level ≥ C7 complete motor (AIS A or B). |
| Population | The subjects are persons with spinal cord injury, neurological level ≥ C7 complete motor (AIS A or B) whose muscles under lesions respond to stimulation since the second motor neuron is spared, these subjects cannot access conventional tendon transfer surgery and are waiting for an alternative technological solution including FES |
| Main Inclusion criteria | 1. Patients who have signed the written consent form to participate in the study after free and informed information  2. Patient affiliated to a social security scheme.  3. Neurological level ≥ C7  4. 18 years old ≤ age ≤ 65 years old,  5. Complete traumatic injury: defined by an A or B score on the AIS scale. (AIS A or B: complete motor deficit under lesion.  6. Neurological stability (no modification of muscle testing)> 6 months,  7. Post-injury duration> 6 months  8. Patients who do not have active muscle resources for conventional tendon transfer surgery in the forearm and hand (with reference to the ICSHT classification <3), |
| Exclusion criteria | 1. Patient deprived of liberty (by judicial or administrative decision).  2. Adult patient subject to a legal protection measure or unable to express consent  3. Participation in another ongoing clinical trial  4. Pregnant or breastfeeding women or women of childbearing potential without effective contraception  5. Spasticity and flexion or extension contractures of the upper limbs of a destabilizing nature.  6. Unstable epilepsy with notion of a seizure occurring within the previous 12 months.  7. Unstable cardiovascular pathology (coronary artery disease, major hypertension, heart failure, etc.).  8. Infectious pathology during treatment during the inclusion visit  9. Wearing a pacemaker.  10. Dermatological problems contraindicating the application of surface electrodes.  11. Body weight> 100 kg  12. Psychiatric illness and / or history contraindicating participation in research  13. Any contraindication to anaesthesia and / or surgery  14. Hypersensitivity to low molecular weight heparin (LMWH) or to one of the excipients of the specialty used  15. History of immune-mediated heparin-induced thrombocytopenia (HIT) in the past 100 days or presence of circulating antibodies  16. Clinically significant active bleeding or condition associated with a high risk of bleeding  17. Electrical mapping identified as negative during the inclusion visit, that is to say with muscles revealing a score <4 MRC for at least one of the extensors (ECRL, ECRB, EDC, EPL ) or one of the flexors (FPL, FDS, FDP). |
| Device under investigation | Neural electrical stimulator triggered by a patient with tetraplegia allowing the restoration of the grip.  Prototype device consisting of two implanted electrodes, an external stimulator and a measuring device.  The measuring system is used within the framework of its CE marking to detect the patient voluntary movements to trigger stimulation.  The electrode-stimulator system, not CE marked, is used for short term (30 days). It is a class IIa device.  A full description of the medical device under investigation was provided by manufacturers to the sponsor in the Investigation Brochure. A compliance to the main ISO standard and the Essential Requirements of the EU Directives 93/42 and 90/385 was followed. |
| Expected benefits for participants and for society | - in the short term, the benefits for the subject can only be observed for the duration of the experiment, the duration of which is 30 days. To enhance their commitment to the protocol, participants will benefit from full medical support - in the medium and long term in a specialized PRM environment, the benefits stem from the promise presented by this experiment for patients with tetraplegia who cannot benefit from functional musculotendinous transfer surgery, to use a device allowing them to regain muscle movements. gripping, usable outside a clinical context, thus contributing to the increase of their autonomy***.*** |
| Risks added by the research | In the short term, these are risks specific to  • any anesthesia and surgery procedure regardless of the surgical procedure  • local postoperative complications (local hematoma, allergy, local sepsis)  • general postoperative complications related to tetraplegia. These are more particularly the so-called decubitus complications  • skin (pelvic pressure ulcer or other site)  • respiratory (bronchopulmonary infection)  • urological (urinary tract infection of the lower or upper apparatus)  • thromboembolism (thrombophlebitis of the lower limbs and pulmonary embolism)  The intraoperative risks associated with percutaneous implantation of neural electrodes are minimal.  Medium and long term: No clinical risk has been identified apart from the psychological risk of experiencing post-explant distress or disillusionment. Psychological support will be systematically offered.  A risk analysis following ISO 14971 was conducted. |
| Investigation Gantt | - Inclusion: 12 months - Participation of patients: 2.5 months   - Device use: 30 days   - Post trial follow-up : 45 days - Total duration: 18 months |
| Process implementation | The selected patient will have been previously identified by the medico-surgical team, as a potential candidate.  a) The selection visit (D-90 to D-30 before implantation of the electrodes) will be carried out by the coordinating investigating doctor, specialist in Physical Medicine and Rehabilitation (Dr Charles FATTAL) who will follow the patient throughout the duration of the 'experimentation. This visit will take place during a consultation.  b) The inclusion visit will be carried out between D-30 to D-1 of the implantation of the electrodes and will involve  ● carrying out muscle mapping ("electrical mapping")  ● signing the consent form  In case of eligibility, will be carried out before implantation  ● analytical and functional tests (excluding stimulation)  ● pre-tests to obtain functional movements by surface electrodes  ● pilot interface / external stimulation pre-tests  c) Clinical follow-up visits will be carried out daily from D0 = day of implantation until D + 29 = day of implantation and will consist of  ● clinical monitoring of general signs  ● clinical monitoring of comorbidities  ● monitoring of local signs around the implantation site  ● routine biological monitoring  ● Anticoagulation for preventive purposes from D0 to D + 15  ● Psychological support if needed  The evaluations specific to the protocol will also be carried out from D + 3 until D + 29 = day of the implantation.  They will associate  ● an analytical and functional assessment,  ● an assessment of tolerance and acceptability,  ● an evaluation of the ordering methods  ● a technical evaluation.  d) The site visit will be carried out on D + 30  e) The end of research visit will be carried out on D + 45 |
| Scheduled inclusions | 6 patients |
| Selected patients | 2 (due to COVID pandemic situation limited to 2 patients) |
| Financial support | KIC EIT Health European Program grant #20682 |
| Ethics committee and competent authorities approvals | The protocol was approved by the French Ethics Committee (CPP Ouest IV Nantes, France, ID-RCB #2019-A00808-49) and the French Health Agency (ANSM). Patients signed an informed consent. The information was given in their native language (French) together with the consent form also in their native language (French). |
